# Differential Gene Expression in the Siphonophore *Nanomia bijuga* (Cnidaria) Assessed with Multiple Next-Generation Sequencing Workflows

**DOI:** 10.1371/journal.pone.0022953

**Published:** 2011-07-29

**Authors:** Stefan Siebert, Mark D. Robinson, Sophia C. Tintori, Freya Goetz, Rebecca R. Helm, Stephen A. Smith, Nathan Shaner, Steven H. D. Haddock, Casey W. Dunn

**Affiliations:** 1 Department of Ecology and Evolutionary Biology, Brown University, Providence, Rhode Island, United States of America; 2 Epigenetics Laboratory, Cancer Research Program, Garvan Institute of Medical Research, Sydney, New South Wales, Australia; 3 Bioinformatics Division, Walter and Eliza Hall Institute of Medical Research, Parkville, Victoria, Australia; 4 Heidelberg Institute for Theoretical Studies, Heidelberg, Germany; 5 Monterey Bay Aquarium Research Institute, Moss Landing, California, United States of America; Centre for Genomic Regulation (CRG), Universitat Pompeu Fabra, Spain

## Abstract

We investigated differential gene expression between functionally specialized feeding polyps and swimming medusae in the siphonophore *Nanomia bijuga* (Cnidaria) with a hybrid long-read/short-read sequencing strategy. We assembled a set of partial gene reference sequences from long-read data (Roche 454), and generated short-read sequences from replicated tissue samples that were mapped to the references to quantify expression. We collected and compared expression data with three short-read expression workflows that differ in sample preparation, sequencing technology, and mapping tools. These workflows were Illumina mRNA-Seq, which generates sequence reads from random locations along each transcript, and two tag-based approaches, SOLiD SAGE and Helicos DGE, which generate reads from particular tag sites. Differences in expression results across workflows were mostly due to the differential impact of missing data in the partial reference sequences. When all 454-derived gene reference sequences were considered, Illumina mRNA-Seq detected more than twice as many differentially expressed (DE) reference sequences as the tag-based workflows. This discrepancy was largely due to missing tag sites in the partial reference that led to false negatives in the tag-based workflows. When only the subset of reference sequences that unambiguously have tag sites was considered, we found broad congruence across workflows, and they all identified a similar set of DE sequences. Our results are promising in several regards for gene expression studies in non-model organisms. First, we demonstrate that a hybrid long-read/short-read sequencing strategy is an effective way to collect gene expression data when an annotated genome sequence is not available. Second, our replicated sampling indicates that expression profiles are highly consistent across field-collected animals in this case. Third, the impacts of partial reference sequences on the ability to detect DE can be mitigated through workflow choice and deeper reference sequencing.

## Introduction

Siphonophores belong to Cnidaria, a diverse group of animals that also includes corals, *Hydra*, and jellyfish. Like a coral, each siphonophore is a colonial organism made up of many genetically identical multicellular zooids (bodies) that arise by asexual reproduction but remain attached and physiologically integrated to each other [1], [2], [3], [4]. Unlike most other colonial animals, where all the zooids are structurally and functionally identical, siphonophore zooids are functionally specialized for particular tasks such as feeding, swimming, defense, or sexual reproduction. To date, there have been no studies of differential gene expression between functionally specialized zooids in siphonophores. Such analyses would help identify genes that specify zooid types, and play a role in the development and functions of different zooid phenotypes.

Next generation sequencing (NGS) has rapidly transformed high-throughput analyses of gene expression [5], [6], [7], [8], [9]. In sequencing-based expression studies, fragments of transcripts are sequenced and the resulting reads are mapped to known gene reference sequences. The number of reads that map to each gene sequence in the reference provides a measure of its expression level [10], [11]. To date, NGS expression studies have been largely limited to model species because their well-annotated genomes provide high quality references for mapping [11], [12]. There is, however, growing interest in using these tools to quantify expression in non-model species.

Several studies taking a variety of approaches along these lines have recently been published. Bellin *et al.* used Roche 454 sequencing to assemble gene reference sequences for the grape vine, *Vitis vinifera*, and microarrays based on these sequences to quantify expression [13]. Fraser *et al.* constructed a gene reference for the guppy, *Poecilia reticulata*, also with Roche 454, but quantified expression with Illumina mRNA-Seq [14]. Other studies have used Illumina mRNA-Seq data rather than Roche 454 to assemble gene references, and tag-based [15] or mRNA-Seq [16] Illumina data to quantify expression. Some of these studies lack biological replication, which makes it difficult to assess the significance of the results. The wide variation in methods across these studies provide interesting glimpses into the benefits and drawbacks of different approaches for measuring expression in non-model organisms, but such comparisons are difficult to interpret across studies since entirely different organisms are under investigation. There is a pressing need for well-replicated expression studies on non-model organisms that use multiple methods to measure expression on the same samples.

In non-model species, reference gene sequences can be derived from the same transcript reads that are used to quantify gene abundance, providing a one-step approach to expression analyses in non-model species. For example, the number of reads in *de novo* assemblies can be used to measure expression [17]. However, one-step reference sequencing and expression quantification is not cost effective for many studies. Assembling raw sequence reads into a reference of gene sequences is best served by long reads [18], but quantifying gene abundance is best served by having many reads [19]. It is less expensive to collect short reads than long reads, so collecting long reads across all the samples to be analyzed (including multiple treatments and biological replicates) would therefore greatly increase the cost of the project or greatly reduce the number of reads that could be sequenced for quantification.

**Figure 1:**
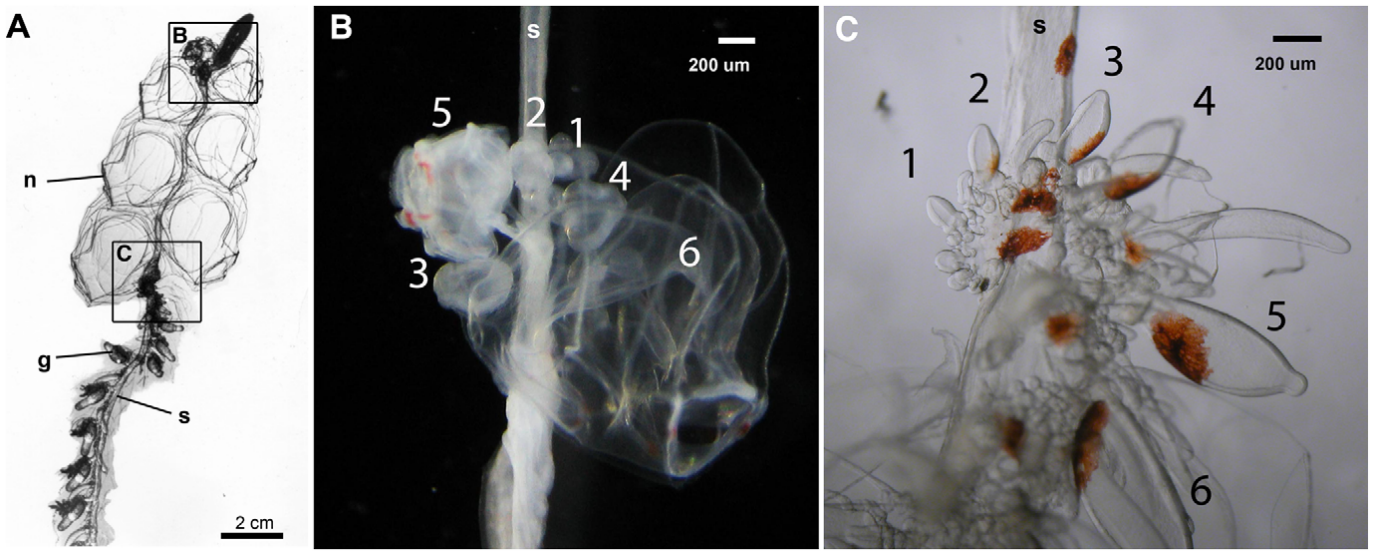
Tissues sampled from the siphonophore *Nanomia bijuga*. (**A**) Paired samples of young nectophores (**B**) and young gastrozooids (**C**) were removed from each of three remotely operated vehicle-collected specimens (see video S1). n: nectophore, g: gastrozooid, s: stem of the colony. Frames in (**A** *)* indicate regions shown in (**B** *)* and (**C** *)*. Numbers indicate the sampled zooids.

Here we use a hybrid strategy that leverages the advantages of long reads for assembling gene predictions and short reads for quantifying transcript abundance. We apply this hybrid long-read/short-read sequencing strategy to investigate differential gene expression between specialized zooids in the siphonophore *Nanomia bijuga* (Figure 1 and Video S1). In this preliminary survey, we focus on two zooid types — developing gastrozooids (feeding polyps) and developing nectophores (swimming medusae).

We used Roche 454 sequencing, with long reads on the order of 400 bp [20], to assemble a partial gene reference dataset. Given the depth of 454 sequencing, some gene sequences are expected to be full length, some to be missing one or both ends, and others to be fragmentary (*i.e.*, different reference sequences may come from different parts of the same gene). To get multiple independent perspectives on the ability to assess differential expression when only a partial reference is available, we collected short-read data from the same samples with three different off-the-shelf expression workflows: SOLiD SAGE (Life Technologies), Illumina mRNA-Seq, and Helicos Digital Gene Expression (DGE). These workflows differ in sample preparation protocols (Figure S1), sequencing platform, and read mapping. All these differences have the potential to impact each workflow's ability to measure differential gene expression.

Both the Helicos and SOLiD sample preparation protocols are tag based – a single read is generated from a particular region of each sequenced mRNA molecule. In the case of Helicos Digital Gene Expression (DGE), the protocol is designed to generate a single read at the 5′ end of each sequenced transcript [21]. In the case of the SOLiD SAGE protocol, the tag site is adjacent to the 3′-most *NIaIII* endonuclease cleavage site [22], [23]. In the case of Illumina mRNA-Seq, the RNA is fragmented and multiple reads are sequenced at random locations along the length of each transcript. The number of mRNA-Seq reads is therefore related to gene length as well as expression [24].

Expression analyses of field-collected specimens, such as the present study, capture expression differences due to variation in genotypes, environmental history, and other factors that can obscure or mislead the analyses of interest (tissue-specific expression in this case) [25]. It is therefore critical to design a sampling strategy that can capture and identify these multiple effects. We collected three replicated pairs of data, where both gastrozooids and nectophores were collected from three different colonies. In contrast to collecting each tissue sample from a different colony, this paired sampling strategy maximized our ability to examine both between-colony effects (e.g., environment, ontogeny, and genotype) and within-colony effects (zooid type) since there are replicate samples of each colony as well as of each tissue type.

This study has implications for the analysis of gene expression in many other taxa. The vast majority of species on earth will never be cultured in the lab, so addressing these important technical issues regarding reference completeness, workflow selection, and variation in field-collected specimens is essential for the use of these methods for most of the diversity of life. Robust analyses of gene expression in field-collected non-model organisms will enable the investigation of a wide range of phenotypes not present in any canonical model organism (including extremophile physiologies and complex lifecycles) and enable densely-sampled evolutionary analyses of gene expression.

## Results

### Reference construction using long-read 454 sequencing

Roche Titanium 454 sequencing produced 589,082 reads (Figure S2A), of which 491,191 passed the Newbler filter. Newbler assembled 315,795 of these reads. The Newbler assembly consists of 9,471 genes (isogroups) which include a combined total of 13,727 contigs. 1,007 of the isogroups had multiple contigs and multiple splice variants (isotigs) consisting of different combinations of contigs. The remaining genes had a single contig comprising a single isotig. 41 of the isogroups had multiple contigs but no isotigs that passed the assembler filters. These isogroups lacking isotigs that pass the assembler filters were excluded from the further analysis. Filtered Newbler singletons (see methods) were assembled by CAP3 into 10,594 contigs. The combined set of CAP3 and Newbler reference sequences served as the reference of gene predictions in the successive short read mapping (Figure S2B). 55.9% of the reference sequences have blastx hits to the non-redundant (nr) NCBI protein database (e-value cutoff of 10^−5^).

Not all ribosomal reads were removed by the Newbler filter. These ribosomal reads were distributed across 90 reference sequences, all of which were excluded from further analyses. A single gene possessed a poly-A that was not trimmed by the assembler as it should have been, and this was excluded to prevent non-specific mapping of short reads.

The final set of reference sequences consisted of 19,925 sequences. Many reference gene sequences were partial. In some cases, multiple reference sequences mapped to different regions of the same gene.

### Short-read sequencing and mapping to the reference

The mean numbers of raw reads for each sampled colony (Table S1) were 66.8±2.0 million for Illumina mRNA-Seq, 139.8±31.1 million for Helicos DGE, and 147.5±25.9 million for SOLiD SAGE. The numbers of reads that passed filter were 58.3±1.4 million for Illumina mRNA-Seq and 63.8±18.0 million for Helicos DGE (SOLiD SAGE reads were not filtered prior to mapping). 26.7% of the Illumina reads which passed filter mapped to a set of selected ribosomal sequences (see methods). The fractions of raw reads that mapped to the reference were 4.7% for Helicos DGE, 27.0% for SOLiD SAGE, and 23.4% for Illumina mRNA-Seq. Count numbers were highly consistent across replicate samples within platforms (top two rows of plots in Figure S3, S4, S5), indicating low sample variation even though the specimens were field-collected.

**Figure 2:**
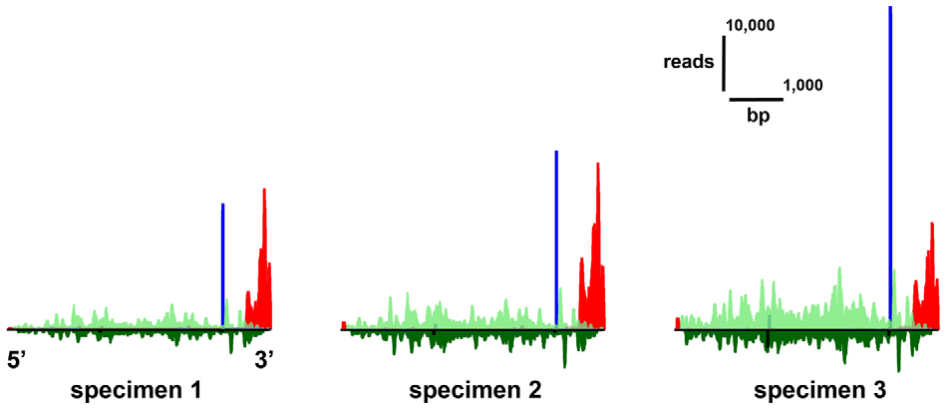
Physical distribution of mapped short-read sequences across an example transcript. Read distribution is shown for a fibrillar collagen (isogroup06489, tblastx e-value 1e-87) for the three nectophore samples. Gastrozooid expression was much lower and not visible on the same scale. All three short-read workflows found significant differential expression for this gene. The gene is drawn in the 5′–3′ direction (4,864 bp). Height of the colored bars indicates the number of reads mapped to that location. Count data are not normalized, so differences in amplitude across samples can be due to differences in sequencing effort across samples. Reads above the line map in the sense direction, below the line in the antisense direction. Helicos DGE reads (red) are sense and unexpectedly tended to map to the 3′ end. Illumina mRNA-Seq reads (green) map to sense and antisense strand along the whole gene. The largest stack of reads for SOliD SAGE (blue) is adjacent to the 3′-most *NlaIII* cutting site.

The physical distribution of mapped reads along the length of reference sequences was consistent for each platform across biological replicates (e.g., Figures 2, S6).

As expected (Figure S1), SOLiD SAGE reads tended to map primarily in stacks adjacent to the *NlaIII* cutting site at the 3′ end of each gene. When mapping to additional sites was observed, these additional stacks were smaller than the primary stacks and generally decreased in count number towards the 5′ end. In some cases there were shadow stacks, sites where multiple reads mapped on the opposite strand on the other side of a *NlaIII* cutting site from the primary stack. These shadow stacks were always smaller than the stack they shadow. Additional and shadow stacks could be the result of incomplete washing following cleavage. They were consistent across replicates, and were therefore not expected to bias expression analyses.

SOLiD SAGE reads mapped to 16,067 of the gene reference sequences. This corresponds to 95.7% of the 16,791 reference sequences with a *NlaIII* site. Given the fragmentary nature of the reference sequences, the presence of a *NlaIII* tag site is not sufficient to determine if the SOLiD SAGE tag site is present. This is because the tag site is adjacent to the 3′-most *NlaIII* site, which could be missing from the reference sequence. Even when the tag site is missing, a gene can still have non-zero counts due to spurious mapping to additional sites.

Illumina mRNA-Seq reads mapped along the full length of reference sequences (Figures 2, S6). The distribution within genes was highly non-uniform, but consistent across biological replicates. It has been suggested that this non-uniform pattern is due to the use of random hexamers to prime cDNA synthesis [26]. This non-uniform pattern could also be due to secondary structure impeding reverse transcription [11]. Illumina reads mapped to 19,534 of the 19,925 (98.0%) gene reference sequences.

Even though the complexity of the dataset made it difficult to describe a global pattern of read distributions, Helicos DGE reads were, contrary to expectations, frequently observed to map to 3′ regions (e.g., Figures 2, S6) and therefore in the same general vicinity as the SOLiD SAGE reads. Helicos Biosciences reported low reverse transcription yield for these samples. If the low yields were due to premature dissociation of the reverse transcriptase, the read location could be displaced towards the 3′ end of the gene as frequently seen here. Helicos DGE reads mapped to 19,485 of the 19,925 (97.7%) gene reference sequences.

### Differential expression – all reference sequences

**Figure 3 pone-0022953-g003:**
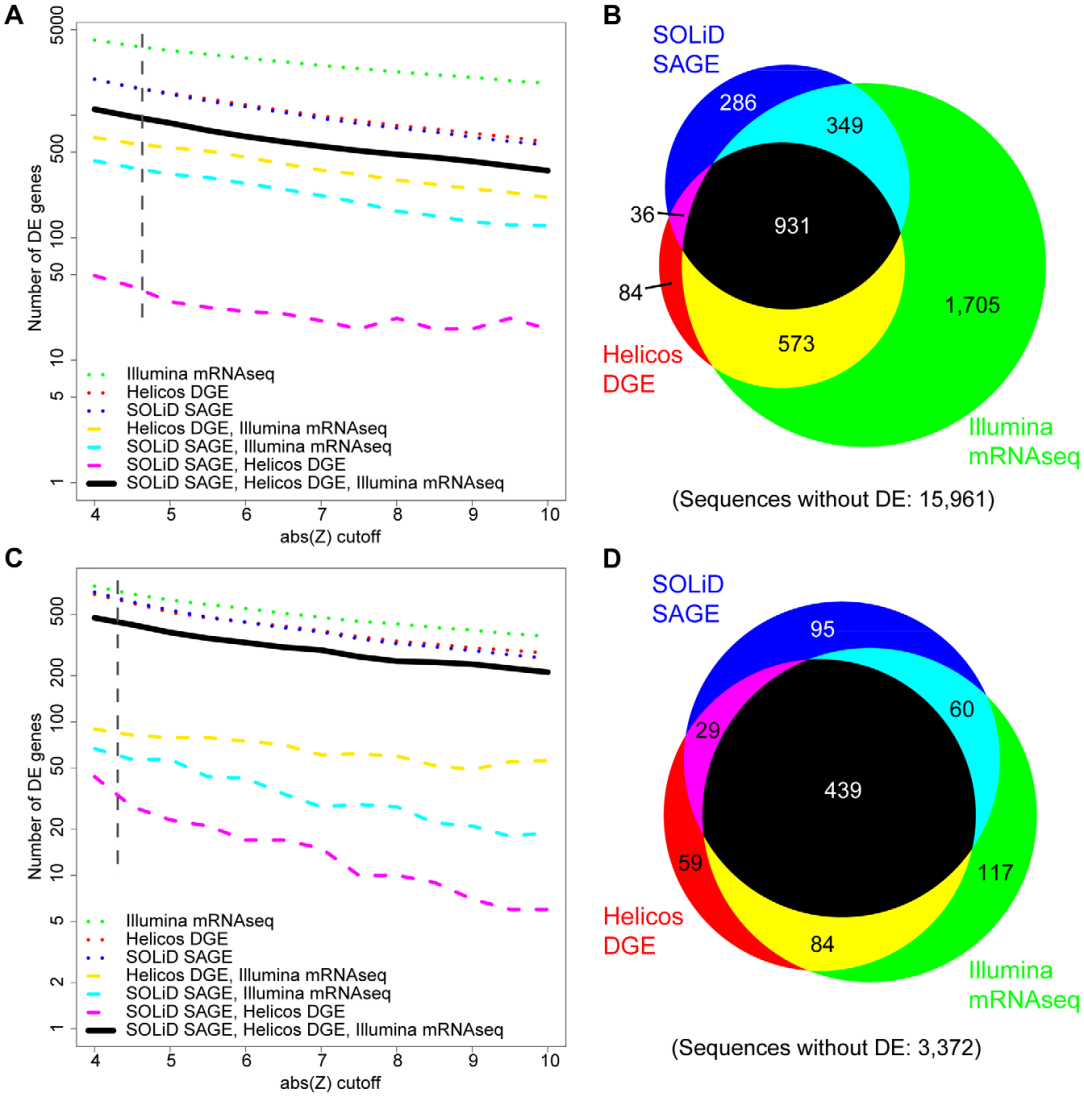
The number and overlap across platforms of reference sequences identified to have differential expression (DE). Analyses of all reference sequences (**A, B**) and analyses of the subset of sequences with the 3′-most *NlaIII* site (**C, D**). (**A, C**) The effect of the Z threshold on the number of genes found to have differential gene expression. The relatively flat lines in all cases indicate little sensitivity to Z threshold. (**B**) Proportional Venn diagram of the number of sequences with significant differential expression (Z>4.71) in analyses of all reference sequences. (**D**) Proportional Venn diagram of the number of sequences with significant differential expression (Z>4.38) in analyses of the subset of sequences with the primary tag site. Areas in Venn diagrams are approximate. Dashed lines in **A,C** indicate Z-values used in **B, D**. The same color code applies to all figures.

A significance threshold of absolute value of Z>4.71, corresponding to a family-wise error rate of 5% (see methods and Figure 3 A, B), was applied in expression analyses of all 19,925 gene reference sequences. The greatest number of significant differentially expressed (DE) reference sequences was identified with Illumina mRNA-Seq (3,558), followed by Helicos DGE (1,624) and SOLiD SAGE (1,602). 931 DE sequences were found by all three workflows (Figure 3B). Gene length (Figure S7) and GC content (not shown) were not significantly related to congruence across platforms. Most DE sequences had higher expression in gastrozooids than nectophores (60.1%, 60.0%, and 70.7% for Illumina mRNA-Seq, SOLiD SAGE, and Helicos DGE) (Figure S7). 79.9% of SOLiD SAGE DE genes and 92.6% of Helicos DGE DE genes were a subset of those identified by Illumina mRNA-Seq (Figure 3B). 200 of the 573 genes with DE on Illumina mRNA-Seq and Helicos DGE but not SOLiD SAGE lacked a *NlaIII* cutting site and were therefore invisible to SOLiD SAGE.

### Differential expression – effects of reference completeness

As each sample preparation protocol generates different read distributions (Figures S1, 2, S6), incomplete gene reference sequences have different impacts on the ability to map reads from each workflow. The completeness of a gene reference sequence is expected to have a roughly linear relationship to the number of mapped mRNA-Seq reads (reads are distributed along the full length of the transcript, so reductions in reference sequence length will proportionally reduce the number of mapped reads), but a threshold effect on tag-based methods (if the tag site is present reads can be mapped, if the tag site is absent they can not be mapped). Mapping efficiency in turn affects the ability of each workflow to detect DE.

We explored the impact of reference sequence completeness on the congruence of DE detection across workflows. We subsampled the set of reference sequences to the 4,255 sequences that unambiguously possess the 3′-most *NlaIII* site (i.e., are complete at the 3′ end and have one or more *NlaIII* sites, see methods) and reassessed DE. When assessing only this subset (Z> 4.38, Figure 3 C, D), there was much broader congruence in the ability to detect DE across all three workflows (Figures 3D, S8). 439 DE sequences were found by all three workflows (Figure 3D).

**Figure 4 pone-0022953-g004:**
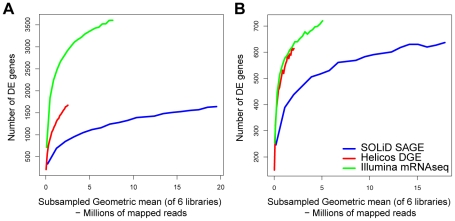
Accumulation curves indicating the number of genes with significant differential expression (DE) when short reads are subsampled. Number of DE sequences are plotted against subsampled library sizes considering the full reference (**A**) and the subset of sequences with the 3′-most *NlaIII* site (**B**). This enables comparison of significance across equivalent library sizes.

These results indicated that missing tag sites in partial reference sequences are a large source of false negatives on SOLiD SAGE and Helicos DGE. When considering all references sequences, the DE accumulation curve for Helicos DGE was intermediate between those of the other two workflows (Figure 4A). When the subset of sequences was considered, Illumina mRNA-Seq and Helicos DGE showed very similar accumulation curves (Figure 4B). The curve for Helicos DGE was steeper at its termination indicating that the workflow would have found a greater number of additional genes with DE with additional sequencing compared to the other workflows. SOLiD SAGE has a shallower accumulation curve regardless of which set of reference sequences was considered (Figure 4 A,B).

### Differential expression – read allocation across genes

**Figure 5 pone-0022953-g005:**
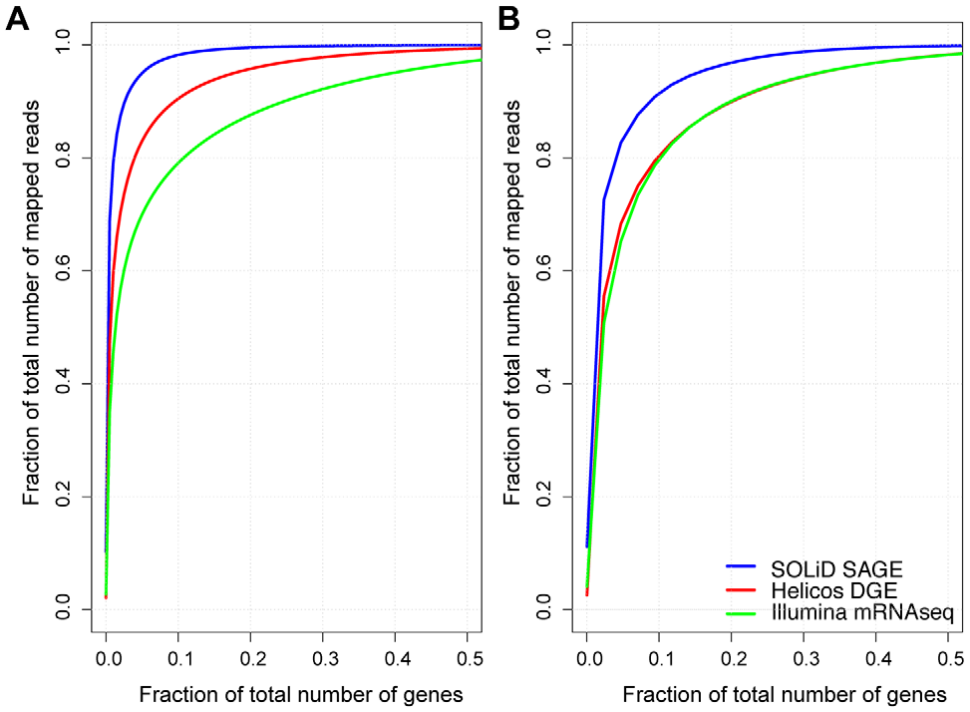
The cumulative fraction of total mapped reads across reference sequences. Fractions of mapped reads are shown for all reference sequences (**A**) and the subset of sequences with the 3′-most *NlaIII* site (**B**). Genes are sorted along the x axis in descending order of the number of mapped reads.

While missing tag sites in incomplete reference sequences account for most differences across workflows, the analyses above indicate that there are additional sources of incongruence in the detection of DE (Figure 3D). One potential additional source of incongruence is differences across workflows in the fraction of mapped reads that went to genes with high expression versus low expression. When considering only the subset of reference sequences that had the 3′-most *NlaIII* site, the 10% of the reference sequences with the most counts account for 79.7% (Illumina mRNA-Seq), 80.3% (Helicos DGE), and 91.4% (SOLiD SAGE) of the total mapped reads (Figure 5).

This indicates that, once reference completeness is accounted for, Illumina mRNA-Seq and Helicos DGE workflows had similar allocations of mapped reads across genes. By comparison, a larger fraction of SOLiD SAGE reads mapped to the most highly expressed genes, and there were proportionally fewer reads that mapped to genes with lower expression (Figure 5A,B). A corresponding read allocation could also be observed when reference sequences with multiple SOLiD stacks were excluded from the analysis (data not shown). These read allocations could be due to Helicos DGE and Illumina mRNA-Seq over-representing genes with low expression, SOLiD over-representing genes with high expression, or some combination of factors. Concordant curves for Illumina mRNA-Seq and the amplification-free Helicos DGE workflow suggested that the pattern is due to overrepresentation of highly expressed genes by the SOLiD workflow. This could potentially be due to PCR overcycling in these particular SOLiD SAGE preparations.

### Consistency across specimens

**Figure 6:**
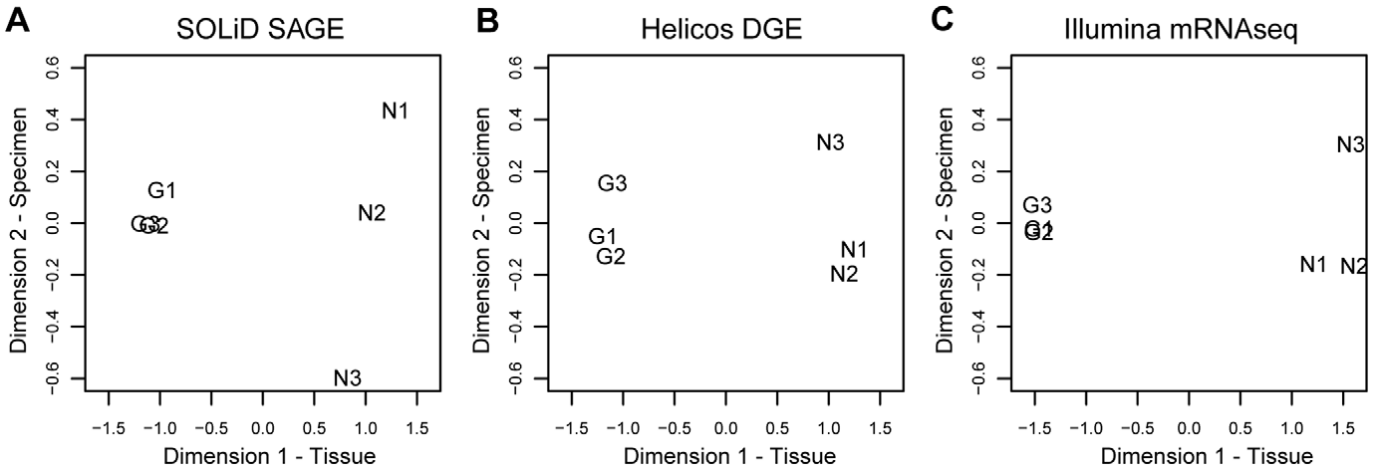
Differential expression across samples from different specimen in different workflows (A–C). Multidimensional scaling plots indicate low degrees of differential expression between samples of the same tissue type originating from different specimen (dimension 2) and higher degrees of differential expression when comparing different tissues types (dimension 1). N: nectophore sample, G: gastrozooid sample. The full set of reference sequences was considered.

Each *Nanomia* specimen was collected independently, had a different genotype, and had a unique environmental history. To the extent that these specimen-specific factors impact gene expression, they could mislead or make it more difficult to detect DE between tissue types [25]. Since our nectophore and gastrozooid samples were paired, with both tissue types sampled from the same three *Nanomia* specimens, we were able to evaluate the impact of specimen-specific factors. The p-values for analyses that include and exclude information on sample pairing were similar, indicating low specimen-to-specimen variability (Figure S9). Differences in p-values in the two analyses were mainly driven by different dispersion estimates for the paired and unpaired analysis. For the complete reference common dispersion estimates were (paired, unpaired): (0.070, 0.144) for SOLiD SAGE, (0.059, 0.094) for Helicos DGE, and (0.040, 0.064) for Illumina mRNA-Seq. In other words, the differences between tissues (Figure 6) were much larger than differences between specimens with nectophore sample 3 being slightly different.

### Characterization of differential expression using *in situ* hybridization

**Figure 7:**
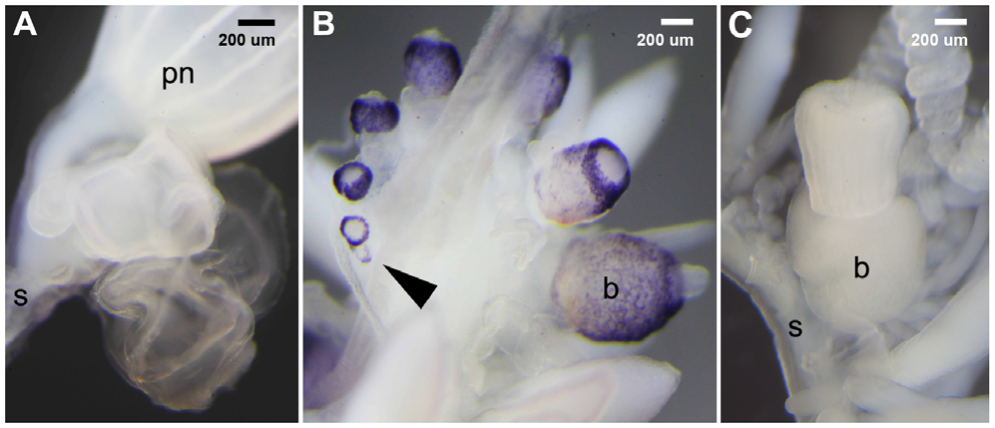
*In situ* characterisation of gene expression. Expression analysis of isogroup03256 in developing nectophores (**A**) and gastrozooids (**B**). The transcript is localized in the basigaster, a region associated with nematogenesis, at the base of gastrozooids and detectable in very early stages of gastrozooid development (arrow). (**C**) Sense control with an unstained basigaster region. *In situ* hybridizations for nectophore specific transcripts have been performed successfully (data not shown). S: stem of the colony, b: basigaster region of the gastrozooid, pn: pneumatophore.

We selected one reference sequence, isogroup03256, for further characterization with *in situ* hybridization. All three workflows found this gene to be strongly expressed in gastrozooids relative to nectophores (log fold change of −9.4 (Illumina mRNA-Seq), −10.7 (SOLiD SAGE) and −10.8 (Helicos DGE)). The *in situ* hybridizations confirmed that expression of this gene is absent in nectophores (Figure 7). They also confirm expression in young gastrozooid buds and revealed that expression in mature gastrozooids is restricted to the basigaster, the region of the gastrozooid where nematocysts (stinging capsules) form [2]. Transcript localization and similarity to domains of a *Hydra magnipapillata* mini-collagen (tblastx, e-value 9e-12) suggested that this high-abundance transcript codes for a protein involved the formation of the nematocyst wall.

## Discussion

There are now a variety of off-the-shelf workflows for analyzing gene expression with next-generation sequencing tools. These workflows are multi-step processes that differ in sample preparation, sequencing methods, and mapping tools. The direct comparison presented here provides the opportunity to examine the degree of congruence between three very different workflows and explore sources of incongruence. We chose workflows that provide the broadest perspective of possible differences, which is critical for comparing studies that use different methods and for selecting workflows for particular applications, at the expense of being able to unequivocally attribute all differences to particular steps within each workflow.

The preparation of our samples for Helicos sequencing gave atypically low cDNA yields and resulted in ectopic tag sites that were frequently observed to be displaced to the 3′. The exact reason for this unexpected outcome for these particular samples could not be determined. The results presented for Helicos here are therefore suboptimal and not typical for the performance of this workflow. Despite problems with sample preparation, however, the Helicos DGE results are still consistent across replicates and congruent with the other two workflows. The data are therefore quite robust to the problems encountered during sample preparation. The detection of ectopic tag sites indicates that it is important to check the physical distribution of mapped reads to verify that library preparation generated the expected products.

Comparative evaluation of read allocation across workflows (Figure 5) suggests overrepresentation of highly expressed genes by the SOLiD SAGE workflow. This could be due to overamplification in this particular sample set, though the use of only eight cycles of amplification suggests that other factors may be at play. The sample preparation kit has since been upgraded and the results presented here might not be indicative of current performance.

The application presented here is typical of that faced by investigators working on non-model organisms – specimens were collected in the field, and the gene reference sequences that were generated are incomplete. We found that the incompleteness of reference sequences explained the greatest fraction of differences between workflows in the ability to detect differential expression (DE). Improving reference completeness is critical to optimizing DE assessment.

The ratio between mapped reads and total number of reads might serve as a rough indicator for the degree of completeness of the transcriptome assembly. In this study 26.8% of the Illumina mRNA-Seq reads, which passed the filter, uniquely map to the gene reference. 26.7% of the reads, which passed the filter, are derived from ribosomal RNA. This leaves 46.5% of reads that do not map uniquely or do not map at all. Reads that do not map at all could be due to several causes, including genetic polymorphism between specimens resulting in multiple mismatches, sequencing errors, genes missing from the reference, and portions of genes missing from the reference (i.e., incomplete gene sequences). However, the fact that highly expressed genes contribute proportionally stronger to the pool of mapped reads complicates the interpretation of the ratio.

The Illumina mRNA-Seq workflow was the least sensitive to gene reference sequence completeness, and identified the greatest number of reference sequences with DE. In this study the tag-based protocols (Helicos DGE and SOLiD SAGE) detected DE for about half as many reference sequences. When only the subset of reference sequences that unambiguously include the 3′-*most NlaIII* site were considered, congruence across platforms was much greater and they all identified a similar set of genes with DE.

Each workflow identified a set of DE reference sequences which the other workflows did not detect (Figure 3D, Illumina mRNA-Seq: 117, SOLiD SAGE: 95, Helicos DGE: 59). We found that different workflows generate different distributions of mapped reads across reference sequences, with SOLiD having fewer reads than the other platforms for genes with low expression (Figure 5A,B). This could explain some of these residual differences in the ability to detect DE. Possible other sources of incongruence could include sequence composition effects leading to lower or higher counts on a particular platform, for example those caused by random hexamer biases [26].

There are at least two important implications of the sensitivity to reference completeness that we identify here. First, as the completeness of gene reference sequences improves, differences between workflows in the ability to detect DE will decrease. Second, when only an incomplete reference sequence is available, mRNA-Seq outperforms tag-based workflows. It is important to note that the decision between tag-based and mRNA-Seq workflows is not a decision between sequencing platforms, as mRNA-Seq sample preparation protocols are available for Illumina, SOLiD, Helicos, and other platforms.

Sequence composition, completeness, and length are properties of the gene reference, and will therefore have the same impact on all samples that are mapped to that reference. However, these reference-specific properties will complicate intergene comparisons, including comparisons between different genes in the same species and orthologs in different species. These challenges apply to some of the most intuitively appealing investigations of the evolution of gene expression, such as the evolution of expression of a gene in a particular tissue across a phylogeny.

In addition to the tissues or treatments under consideration, gene expression is also a function of environmental factors and of the genotype of the sampled organisms [25], [27]. Because we collected three pairs of nectophore and gastrozooid samples from three specimens, we were able to take into account the impact of differences across samples as well as differences between tissues when assessing differential expression. These analyses indicate that expression was highly consistent across specimens. This is consistent with the very low common dispersion in expression for this study. These results also indicate consistent mRNA harvest and high technical reproducibility for each sequencing workflows.

The hybrid design employed here, wherein long-read data are used to generate reference sequences and short-read data are used to quantify gene expression, provides a cost-effective strategy for analyzing differential gene expression in non-model organisms. With growing interest in comparative and ecological functional genomics, such studies will be increasingly common.

## Materials and Methods

### Sampling of *Nanomia bijuga*

*N. bijuga* specimens were collected in Monterey Bay, California, and adjacent waters on May 30, 2009, via blue-water diving from a depth of 10–20 m and on December 11–14, 2009 by ROV Doc Ricketts (R/V Western Flyer) at depths ranging from 200–600 m.

### 454 sequencing

Mature bracts and nectophores were removed and discarded from the intact *Nanomia bijuga* colonies. Siphosomal and nectosomal growth zones, including gastrozooid and nectophore buds, were excised and flash frozen in liquid nitrogen. These growth zones include a broad set of zooids in various stages of development. Total RNA was isolated with TRIzol (Invitrogen) followed by RNeasy (Qiagen) cleanup including DNaseI digestion. Four libraries were prepared for sequencing with 454 GS FLX Titanium chemistry (Roche). Different protocols were used in consecutive library preparations to optimize overall coverage. The first library was prepared with a modified template switching protocol (based on the SMART cDNA Library Construction Kit, Clontech) using SuperScript II (Invitrogen 18064-014) [28]. and pooled total RNA from growth zones of three different animals. 3′ and 5′ adapters containing *SfiI* cutting sites were added during first strand synthesis. (5′ first strand synthesis primer - AAG CAG TGG TAT CAA CGC AGA GTG GCC ACG AAG GCC rGrGrG, 3′ first strand synthesis primer - ATT CTA GAG GCC ACC TTG GCC GAC ATG TTT TCT TTT CTT TTT TTT TCT TTT TTT TTT VN). Primary PCR amplification of the library was conducted on a qPCR thermal cycler with a 5′ PCR primer (AAG CAG TGG TAT CAA CGC AGA GT) and the 3′ synthesis primer. A control reaction was spiked with 1 x SYBR green (Invitrogen S7563) to monitor for overcycling. Primary PCR reactions were purified using the Qiaquick purification kit (Qiagen) and cDNA was quantified with Qubit dsDNA BR (MP 32850). The primary PCR product was diluted (1∶10) and used in a secondary PCR (10 cycles) to generate the required amount of cDNA for 454 sequencing (>10 µg). Secondary PCR product was purified using Qiaquick PCR cleanup kit (Qiagen) and subsequently digested using enzyme *Sfi1* (NEB# R0123L) or enzymes *Sfi1*/*Mme1* (NEB#R0637L) in a double digest (see below). Products were size selected using Chromaspin TE-400 columns (Clontech#636076), blunted using NEB kit (NEB# E1201L), and quantified.

The second library was prepared like the first, except that the 3′ adapter was modified to include a *MmeI* site (PD243Mme-30TC - ATT CTA GAG CGC ACC TTG GCC TCC GAC TTT TCT TTT CTT TTT TTT TCT TTT TTT TTT VN). This adapter was also used in the PCR amplification. Cleavage at this site after library amplification removed most of the poly-A tail. The first and the second library were each sequenced on a quarter of a Roche 454 Titanium plate (EnGenCore, Columbia, SC).

For the third library, total RNA from the nectosomal and siphosomal growth zones of a single specimen was extracted as described above. mRNA was enriched with one round of purification on MPG Streptavidin Complex (Purebiotech). mRNA was sent to Roche 454 Life Sciences (Connecticut) where a cDNA library was prepared with the standard 454 cDNA library preparation protocol and sequenced on a eighth of a Roche 454 Titanium plate.

A fourth library was prepared and sequenced in the same way as the third library, but it was derived from two specimens using mRNA from developing nectophores and gastrozooids.

### Assembly of the transcriptome reference

A two-stage assembly was employed, whereby the singletons from the first assembler (Newbler version 2.3) were assembled with a second assembler (CAP3 version 0.990329). This two-step strategy was suggested by Roche 454, as a large fraction of reads not assembled by Newbler version 2.3 can be assembled by CAP3. Newbler 2.3 explicitly accommodates splice variation, and generates contigs (roughly corresponding to exons), isotigs (which correspond to transcript splice variants), and isogroups (which correspond to genes). The sff files from the multiple runs were combined with the sfffile command and assembled with runAssembly using the -cdna and -nosplit flags, along with a vector file that included all oligonucleotides used in library preparation as well as *Nectopyramis* 28S sequence (Genbank AY026377.1), *N. bijuga* 18S (Genbank AF358071.1), and *Hydra* ribosomal RNA sequences (*Hydra* AEP_28S_18S 9,568 bp, *Hydra* AEP 28S 3,493 bp, *Hydra* AEP 18S 1,800 bp, sequences were provided by G. Hemmrich). A known bug in Newbler 2.3 (Roche, pers. comm.) results in incorrectly split contigs, with some contigs containing sequence from adjacent contigs within isotigs. This was corrected by trimming the contigs prior to mapping (Illumina) or subsampling files of mapped reads (SOLiD); Helicos mapping was unaffected by this bug since it was based on isotig sequences.

Fasta sequence and quality files were generated for the singletons that were not assembled by runAssembly (as identified in the 454 ReadStatus.txt file). Adapters were trimmed with regular expressions and cross_match (vers.0.990329). These trimmed singletons were then assembled with CAP3 (vers. 10/15/07, with the options -z 1 -y 100). Reads that were not assembled by either assembler were not considered further. The Newbler and CAP3 assemblies were pooled and served as the reference sequence for all downstream analyses.

### Sampling strategy and mRNA preparation for short-read sequencing

Three *Nanomia bijuga* specimens were collected by remotely operated underwater vehicle. The live specimens were kept in the dark at 4°C for no more than 14 h before they were processed. Animals were anaesthetized by adding 4°C isotonic magnesium chloride (7.5% MgCl_2_⋅6H_2_0 in distilled water) to about 1/3 of the total volume, large nectophores and bracts were discarded, and the specimens were pinned out in a petri dish lined with Sylgard 184 (Dow Corning). Sampling started immediately after pinning. Sharpened, fine-tipped forceps were used to pluck nectophores and gastrozooids from the stem of the colony. Cryovials were pre-frozen in liquid nitrogen and were stored in liquid nitrogen in between repetitive sampling into the same vial. At sea, samples were kept in liquid nitrogen. On shore they were kept at −80°C and shipped on dry ice.

Young nectophores and gastrozooids were dissected from three different animals (specimen S1–S3), generating three paired tissue samples (Figure 1). First, a series of non-functional developing nectophores was sampled from each of the nectosomal growth zones. The very youngest nectophores were sampled as a cluster (indicated by the 1 in Figure 1B) followed by sampling of the next 4–5 larger nectophores (Figure 1B, 2–6). Following this, a series of the first 5–6 non-functional developing gastrozooids was sampled from the siphosomal growth zone starting with the smallest unambiguously identifiable gastrozooid which could be sampled (Figure 1C). Developing tentacles at the base of the larger gastrozooids were removed before freezing.

mRNA was extracted directly from tissue (New England Biolabs, #S1550S). Samples were thawed on ice after adding 500 µl of lysis/binding buffer, transferred to a homogenization tube and homogenized using a sterile pestle. Samples were added to 100 µl equilibrated Oligo d(T)25 beads. mRNA was eluted in 100 µl elution buffer. After precipitation, using 1/10 volume sodium acetate (3M, pH 5.5) and 2.5 volumes EtOH (overnight), pellets were resuspended in 11 µl water. Isolations yielded 534–785 ng poly-A-enriched mRNA. mRNA integrity was checked on an Agilent Bioanalyzer using the RNA 6000 Pico Kit.

The same set of RNA samples was used for all short read sequencing.

### Helicos sequencing and count generation

mRNA of each sample (150 ng) was sent to Helicos Biosciences, Cambridge, for library preparation and sequencing, which followed standard protocols [21]. Reverse transcription resulted in average cDNA mass of 7 ng per sample corresponding to 5% of the expected cDNA yield (pers. comm. Helicos Biosciences). Samples were sequenced in 17 HelioScope channels on two independent runs. Helicos DGE reads were mapped to isotig (gene isoforms) and cap3 contig sequences. The reference was provided to Helicos Biosciences, who returned sequence data, alignments to the reference, and counts for each gene in the reference (Helisphere-1.2.657 and TranscriptCount 1.2.0). The mean length of mapped reads was 31.9 bp (Helicos read length is dependent on sequence composition and varies from read to read). As 454 sequencing and assembly was not directional, short reads of all platforms were mapped to the forward and the reverse strand of the reference. Multiple mappings of a read to several isotigs (gene isoforms) within an isogroup were collapsed into one to generate isogroup counts.

### SOLiD sequencing and count generation

mRNA of each sample (150 ng) was provided to Life Technologies (Beverly, MA) for SAGE library preparation and sequencing on a SOLiD 3+ instrument. SAGE libraries required 8 cycles of amplification. The reference derived from 454 data was provided to Life Technologies. A virtual reference sequence was generated from the 454 reference by taking 27 bp flanking each of the *NlaIII* sites and concatenating the sequences together. The first 21 bp of each read were mapped to both forward and reverse strand of the virtual reference with the SOLiD Corona Lite pipeline. Life Technologies then returned sequence data and alignments to the reference. We generated unique gene counts by excluding reads that mapped to contigs of more than one gene. Reads mapping to several contigs within an isogroup were only counted once. Only reads that mapped with two or less colorspace mismatches were considered.

### Illumina sequencing and count generation

We prepared libraries for each sample with the Illumina mRNA-Seq sample kit (#RS-930-1001, Illumina Inc.) according to the manufacturer's instructions (versions 09/09). mRNA starting material varied between 138–307 ng (S1_necto: 145 ng, S1_gastro: 138 ng, S2_necto: 159 ng, S2_gastro: 189 ng, S3_necto: 139 ng, S3_gastro: 307 ng). cDNA templates were enriched with 15 cycles of amplification. 85 bp single end reads were sequenced on an Illumina GAIIx according to standard protocols. Basepairs 2–33 of each read were aligned to the contig reference using Casava 1.6, allowing up to two mismatches. Unique gene counts were generated using the output file s_N_sorted.txt file, which contains reads which passed purity filtering and have a unique alignment in the reference. *Nanomia bijuga* ribosomal sequences (18S, 28S, 16S) were added to the 454 reference in order to measure rRNA content within the Illumina mRNA-Seq libraries.

### Data availability

All sequence data have been deposited at the NCBI Short Read Archive (Helicos: accession #SRA028279.1, from 454, Illumina, and SOLiD instruments: accession #SRA027226.2). The count file (File S1) containing expression data for each gene and the gene sequence reference (File S2) are available as supplemental files.

### Statistical testing

We assessed the significance of differential gene expression with edgeR [29], version 2.0.5 according to standard protocols outlined in the package manual. These analyses were run in R version 2.12.2 and analyses for each sequencing workflow were conducted separately. To account for differences in sequencing effort and proportionality across libraries, count data were first normalized by TMM [30] with the calcNormFactors() function. Cox-Reid common dispersions were calculated with the estimateCRDisp() function. A generalized linear model (GLM) was then fit to the data with the glmFit() function, and p-values were calculated using a likelihood ratio test (LRT) with the glmLRT() function. Z values for the congruence analysis (Figures 3, S7, S8) were calculated for each gene by back transforming the LRT p-value onto the standard normal distribution and giving it a sign according to the direction of the change (i.e. Z =  {u: Prob(|X| >u) = p-value/2} x sign(log-fold-change) where X is the standard normal distribution). Positive values of Z indicate higher expression in nectophores than gastrozooids. The threshold for evaluating significance was obtained by applying a Bonferroni correction for multiple tests to a p-value of 0.05 (i.e., dividing 0.05 by the number of genes in the reference) and then calculating the corresponding Z-value as above. For analyses of all reference sequences, the significance threshold is an absolute Z-value >4.707364, and for analyses of only those reference sequences that have a primary tag site the threshold is an absolute Z-value >4.382159. Gene length was calculated as the sum of all contigs in an isogroup.

The GLM was fit in two ways: first, with specimen-specific effects, in addition to an effect for the difference between gastrozooids and nectophores (denoted "paired"); and second, with a common intercept term as well as the difference between tissues (denoted "unpaired"). The p-values comparing these two approaches are shown in Figure S9. Multidimensional scaling plots (Figure 6) were generated using the plotMDS.dge() function.

### Identification of SOLiD tag sites in the gene references

The SOLiD tag site is anchored to the 3′-most *NlaIII* site in the transcript (Figure S1). The absence of an *NlaIII* site definitively indicates that no SOLiD reads can map to a given gene reference sequence. The presence of one or more *NlaIII* tag sites in a reference sequence, though, is not alone sufficient to guarantee that the SOLiD tag site is present in the reference. This is because the 3′ most site may still be absent from the reference sequence. If a reference sequence is complete at the 3′ end and one or more *NlaIII* sites are present, then the SOLiD tag site is present. We assessed the 3′ completeness of our reference sequence by searching for uncleaved 3′ library adapters in raw 454 reads. This allowed us to categorize genes according to SOLiD tag site being absent (no *NlaIII* site: 3,134 genes), tag site present (with both an *NlaIII* site and reads with a 3′ adapter: 4,255 genes), and tag site unknown (with an *NlaIII* site but no reads with a 3′ adapter: 12,536 genes).

### *In situ* Hybridization

Whole mount *in situ* hybridization was carried out as described previously [31]. Dig-labeled riboprobes (716 bp) were prepared for isogroup03256 with the MEGAscript Sp6/T7 kits (AMBION) according to the manufacturer's instructions and using sequence specific primers Fw-CGT ATT CTT TGC CGT CAT TGG C and Rev-GAT CGT ATT TAT GCC GGT GTC CA. Hybridization occurred for 35 h at 60°C. Before detection, tissue was blocked using MAB-B (1x MAB, 1% BSA) for one hour and for two hours in 80% MAB-B/20% heat inactivated sheep serum.

## Supporting Information

Figure S1 **Overview of sample preparation procedures for the three short-read sequencing protocols used to quantify transcript expression.** The Digital Gene Expression protocol (Helicos) and the SAGE protocol (SOLiD) generate a single sequencing read (tag) from a particular region of each sequenced RNA molecule. The mRNA-Seq protocol (Illumina) generates multiple reads per sequenced mRNA molecule, spread across the length of the transcript, since mRNA is fragmented and then randomly primed. The circles in the SOLiD SAGE protocol indicate beads.
(PNG)

Figure S2 ***De novo*** **transcriptome assembly using 454 sequencing.** Overview of the assembly process for 454 data, including the number of sequences at each step (**A**). The distribution of the reference sequence length (**B**). For isogroups with multiple isotigs, gene length was calculated as the sum of the length of all contigs.
(PNG)

Figure S3 **Correlation of gene expression quantification using Helicos DGE.** The top row shows the three pairwise correlations of counts per reference sequence between the three nectophore samples. The middle row shows the pairwise correlations between the three gastrozooid samples. The bottom row shows the correlation between pairs of nectophore and gastrozooid samples.
(PNG)

Figure S4 **Correlation of gene expression quantification using SOLiD SAGE.** The top row shows the three pairwise correlations of counts per reference sequence between the three nectophore samples. The middle row shows the pairwise correlations between the three gastrozooid samples. The bottom row shows the correlation between pairs of nectophore and gastrozooid samples.
(PNG)

Figure S5 **Correlation of gene expression quantification using Illumina mRNA-Seq.** The top row shows the three pairwise correlations of counts per reference sequence between the three nectophore samples. The middle row shows the pairwise correlations between the three gastrozooid samples. The bottom row shows the correlation between pairs of nectophore and gastrozooid samples.
(PNG)

Figure S6 **Mapped read distribution across selected transcripts.** Read distribution is shown for ten different reference sequences **(A–J)** and all replicates (necto 1–3, gastro 1–3). Sequences are orientated in 5′ to 3′ direction. The physical distributions of mapped reads (non-normalized counts) across reference sequences were consistent for each platform across biological replicates. The given examples support the view of not exclusive but frequent ectopic read mapping of Helicos DGE reads to the 3′ end of the reference sequences. In each plot reads above the line map in the sense direction, below the line in the antisense direction. Helicos DGE reads (red) map to the sense strand, Illumina mRNA-Seq reads (green) map to sense and antisense strands along the whole reference sequence. The largest stack of reads for SOliD SAGE (blue) is adjacent to the 3′-most *NlaIII* cutting site. Height of the colored bars indicates the number of reads mapped to that location. 454 coverage at each nucleotide position of the reference sequence is shown in the lower part of each plot (scale bar indicates maximum depth of coverage in numbers of 454 reads). Best blast hit (tblastx against NCBI nr database) and respective e-value (e-value cutoff of 10^−5^) are given for each reference sequence if available. Plots in **A–E** show read distributions for nectophore specific transcripts and plots in **F–J** gastrozooid specific transcripts. Plots in **A** show read distributions (all replicates and both tissue types) for the fibrillar collagen (isogroup06489) also presented in Figure 2. Plots in **F** show read distributions for isogroup03256 (numbers of mapped SOLiD reads were downscaled by a factor of 100) which was further characterized by *in situ* hybridization (Figure 7).
(PDF)

Figure S7 **Congruence in detection of DE across sequencing workflows, considering all sequences in the reference.** Visualized are scatterplots of pairwise comparisons (significance cuttoff Z>4.707364) (left), the frequency of DE in nectophores and gastrozooids for each category (middle) and DE in dependency of gene length. Positive Z values represent higher expression in nectophores, compared to gastrozooids (**A**) Helicos DGE – Illumina mRNA-Seq. (**B**) SOLiD SAGE – Illumina mRNA-Seq. (**C**) SOLiD SAGE – Helicos DGE. N: sequences indicated to be differentially expressed in nectophores, G: sequences indicated to be differentially expressed in gastrozooid, T: size distribution of all reference sequences.
(PNG)

Figure S8 **Congruences in detection of DE across sequencing workflows, considering the subset of reference sequences with the 3**′**-most** ***NlaIII*** **site.** Visualized are scatterplots of pairwise comparisons (significance cutoff Z>4.382159) (left), the frequency of DE in nectophores and gastrozooids for each category (middle) and DE in dependency of gene length. Positive Z values represent higher expression in nectophores, compared to gastrozooids (**A**) Helicos DGE – Illumina mRNA-Seq. (**B**) SOLiD SAGE – Illumina mRNA-Seq. (**C**) SOLiD SAGE – Helicos DGE. N: sequences indicated to be differentially expressed in nectophores, G: sequences indicated to be differentially expressed in gastrozooid, T: size distribution of all reference sequences.
(PNG)

Figure S9 **Variability of gene expression across field collected specimen of** ***Nanomia bijuga*** **.** Comparison of likelihood ratio test p-values with or without considering that the samples are paired. Consistent p-values indicate low specimen-specific effects.
(PNG)

File S1 **Countfile.** Tab-delimited text file with read counts and other relevant data for each sequence in the reference. A complete list of file contents is provided within the file.
(TXT)

File S2 **Gene reference (in FASTA format).** Contigs (fasta format) of both the Newbler and the CAP3 assembly of 454 reads which were used for mapping the short reads. Unique counts of Newbler contigs belonging to the same isogroup were summed up to generate isogroup counts.
(FASTA)

Table S1 **Sequencing statistics for the three short-read platforms used to quantify gene expression in** ***Nanomia bijuga*** **.** Shown are mean values and standard deviations for read numbers (in million reads) collected from three different animals. Percentage of mapped reads was calculated relative to raw reads.
(PNG)

Video S1 ***In situ*** **observations of the siphonophore** ***Nanomia bijuga*** **, and sampling procedure using a remotely operated underwater vehicle.** In Monterey Bay *Nanomia bijuga* can be found from surface waters down to a depth of 700 m. This video was taken at a depth of 612 m.
(MOV)
