# Supplementary figures and images for: Differential Gene Expression in the Siphonophore Nanomia bijuga (Cnidaria) Assessed with Multiple Next-Generation Sequencing Workflows
Source: PLoS One. 2011 Jul 29;6(7):e22953. doi: 10.1371/journal.pone.0022953 (PMC3146525; doi:10.1371/journal.pone.0022953)

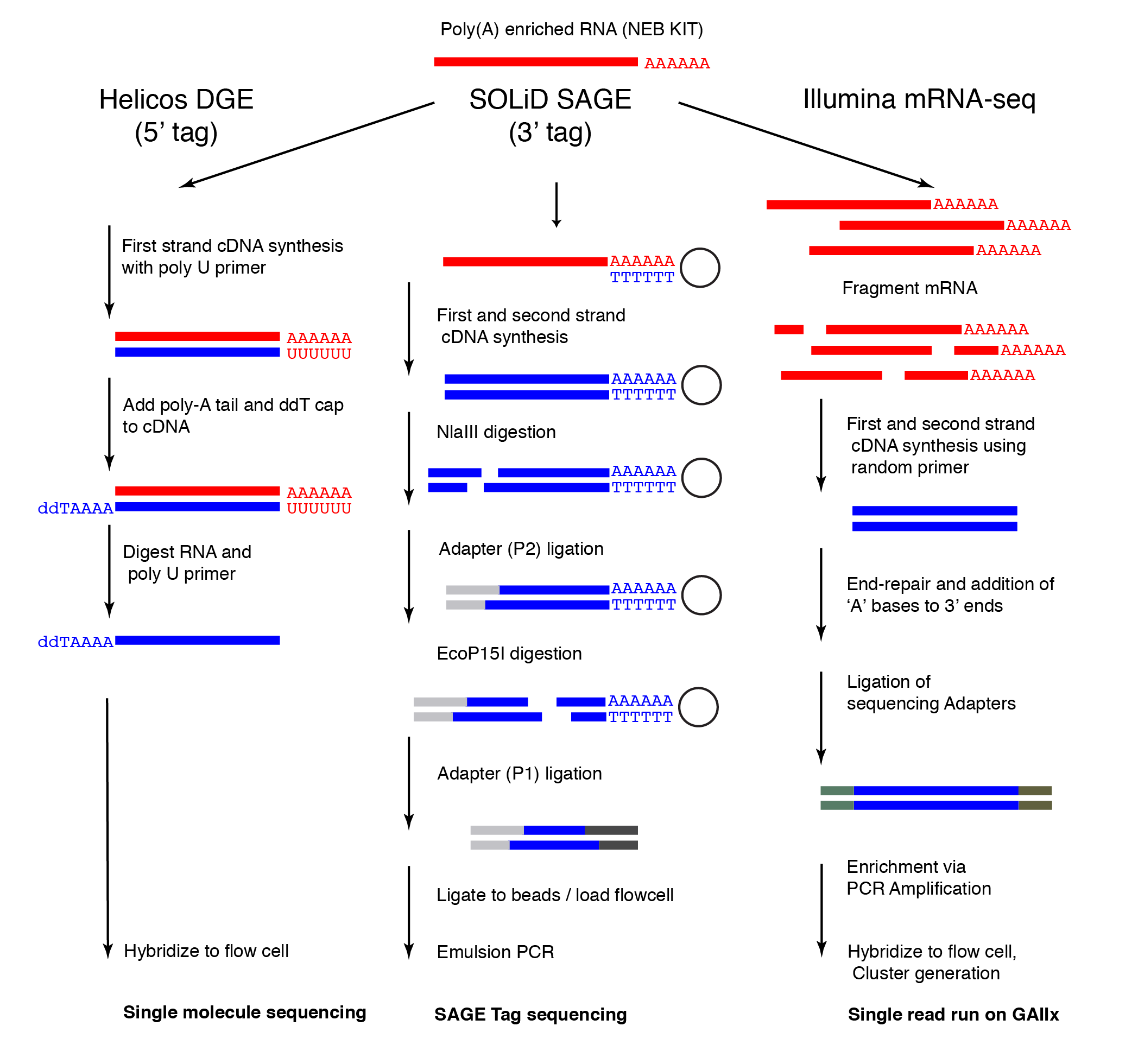

Supplement: Figure S1 — Overview of sample preparation procedures for the three short-read sequencing protocols used to quantify transcript expression. The Digital Gene Expression protocol (Helicos) and the SAGE protocol (SOLiD) generate a single sequencing read (tag) from a particular region of each sequenced RNA molecule. The mRNA-Seq protocol (Illumina) generates multiple reads per sequenced mRNA molecule, spread across the length of the transcript, since mRNA is fragmented and then randomly primed. The circles in the SOLiD SAGE protocol indicate beads. (PNG) [file pone.0022953.s001.png]

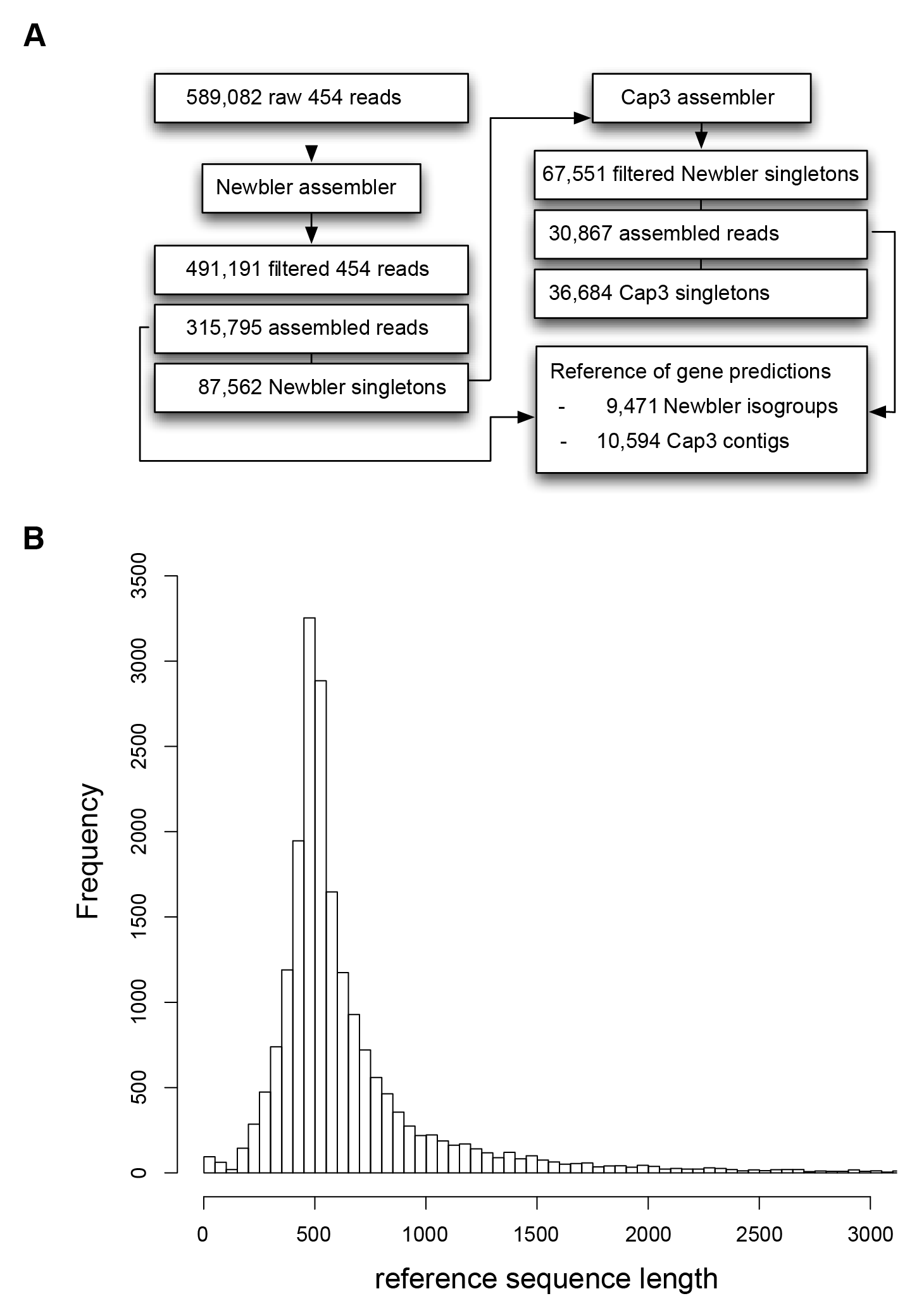

Supplement: Figure S2 — De novo transcriptome assembly using 454 sequencing. Overview of the assembly process for 454 data, including the number of sequences at each step (A). The distribution of the reference sequence length (B). For isogroups with multiple isotigs, gene length was calculated as the sum of the length of all contigs. (PNG) [file pone.0022953.s002.png]

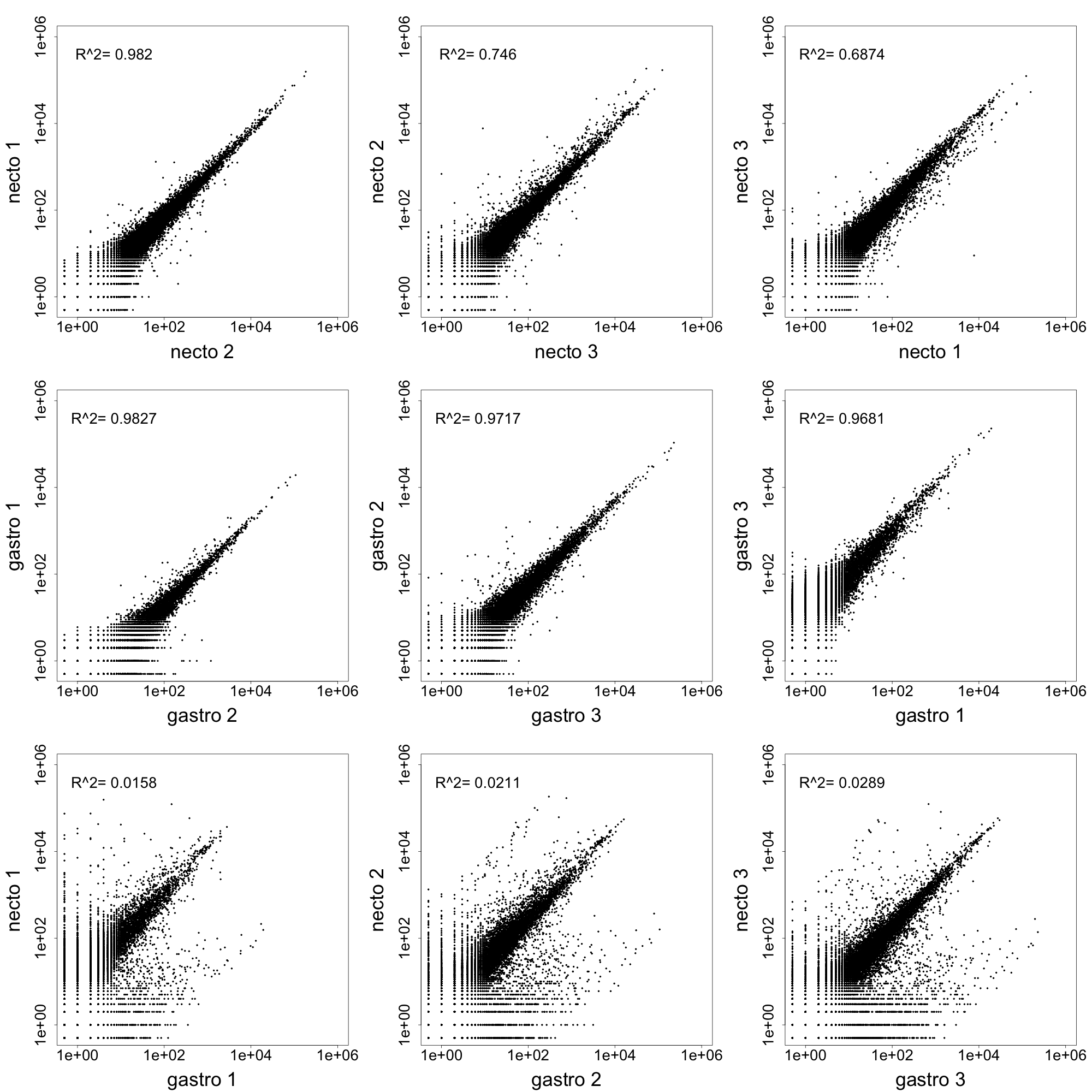

Supplement: Figure S3 — Correlation of gene expression quantification using Helicos DGE. The top row shows the three pairwise correlations of counts per reference sequence between the three nectophore samples. The middle row shows the pairwise correlations between the three gastrozooid samples. The bottom row shows the correlation between pairs of nectophore and gastrozooid samples. (PNG) [file pone.0022953.s003.png]

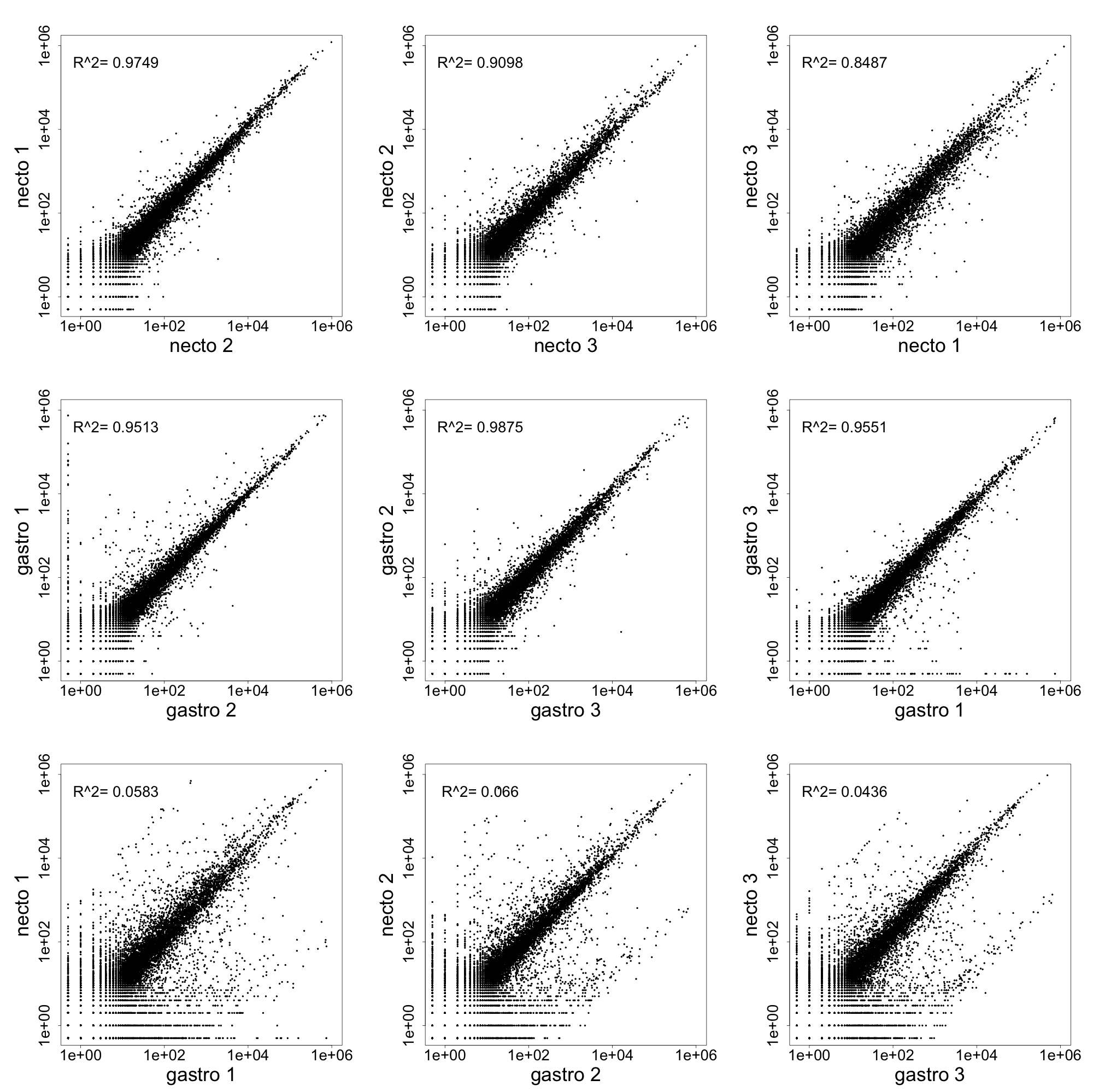

Supplement: Figure S4 — Correlation of gene expression quantification using SOLiD SAGE. The top row shows the three pairwise correlations of counts per reference sequence between the three nectophore samples. The middle row shows the pairwise correlations between the three gastrozooid samples. The bottom row shows the correlation between pairs of nectophore and gastrozooid samples. (PNG) [file pone.0022953.s004.png]

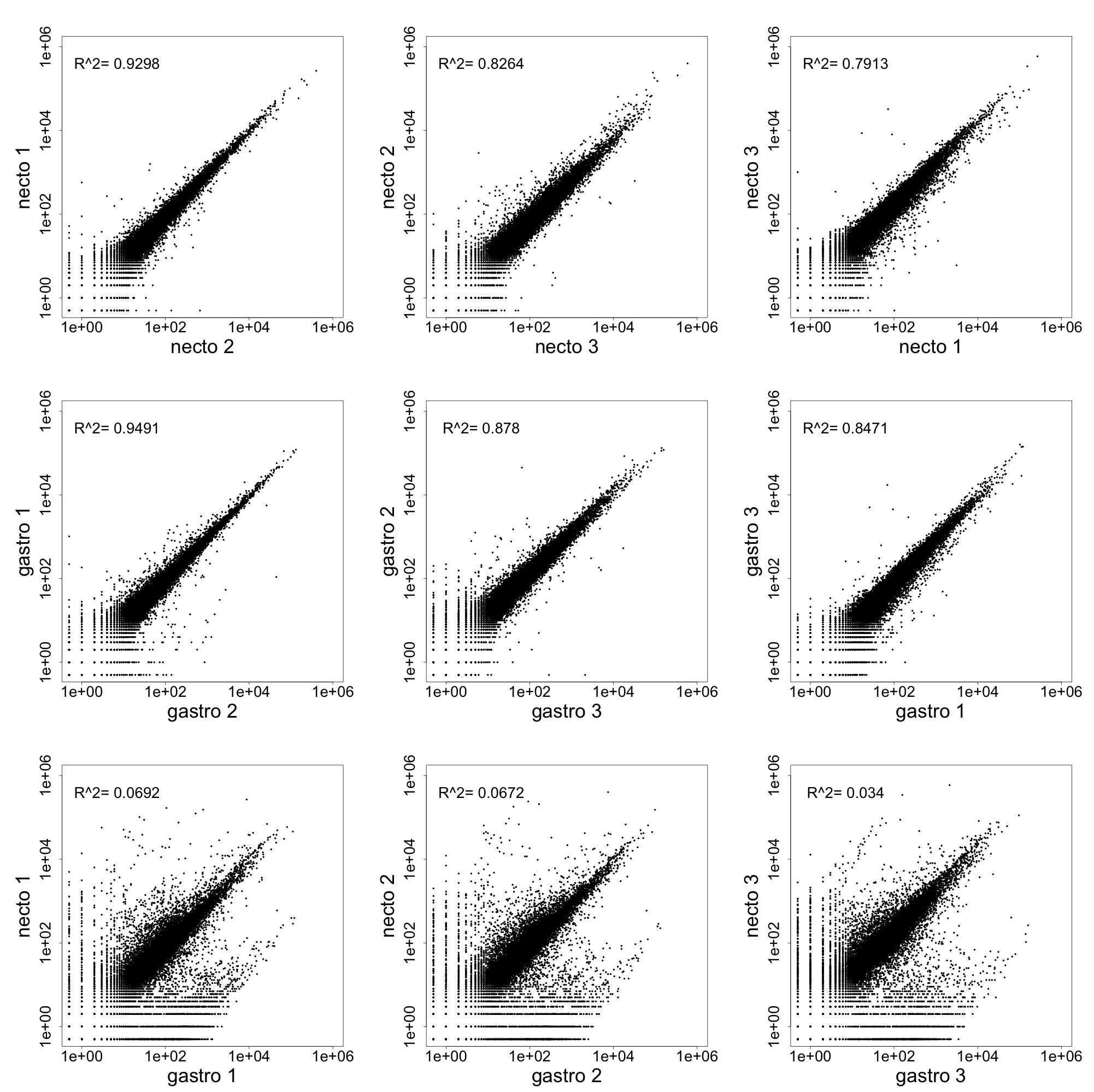

Supplement: Figure S5 — Correlation of gene expression quantification using Illumina mRNA-Seq. The top row shows the three pairwise correlations of counts per reference sequence between the three nectophore samples. The middle row shows the pairwise correlations between the three gastrozooid samples. The bottom row shows the correlation between pairs of nectophore and gastrozooid samples. (PNG) [file pone.0022953.s005.png]

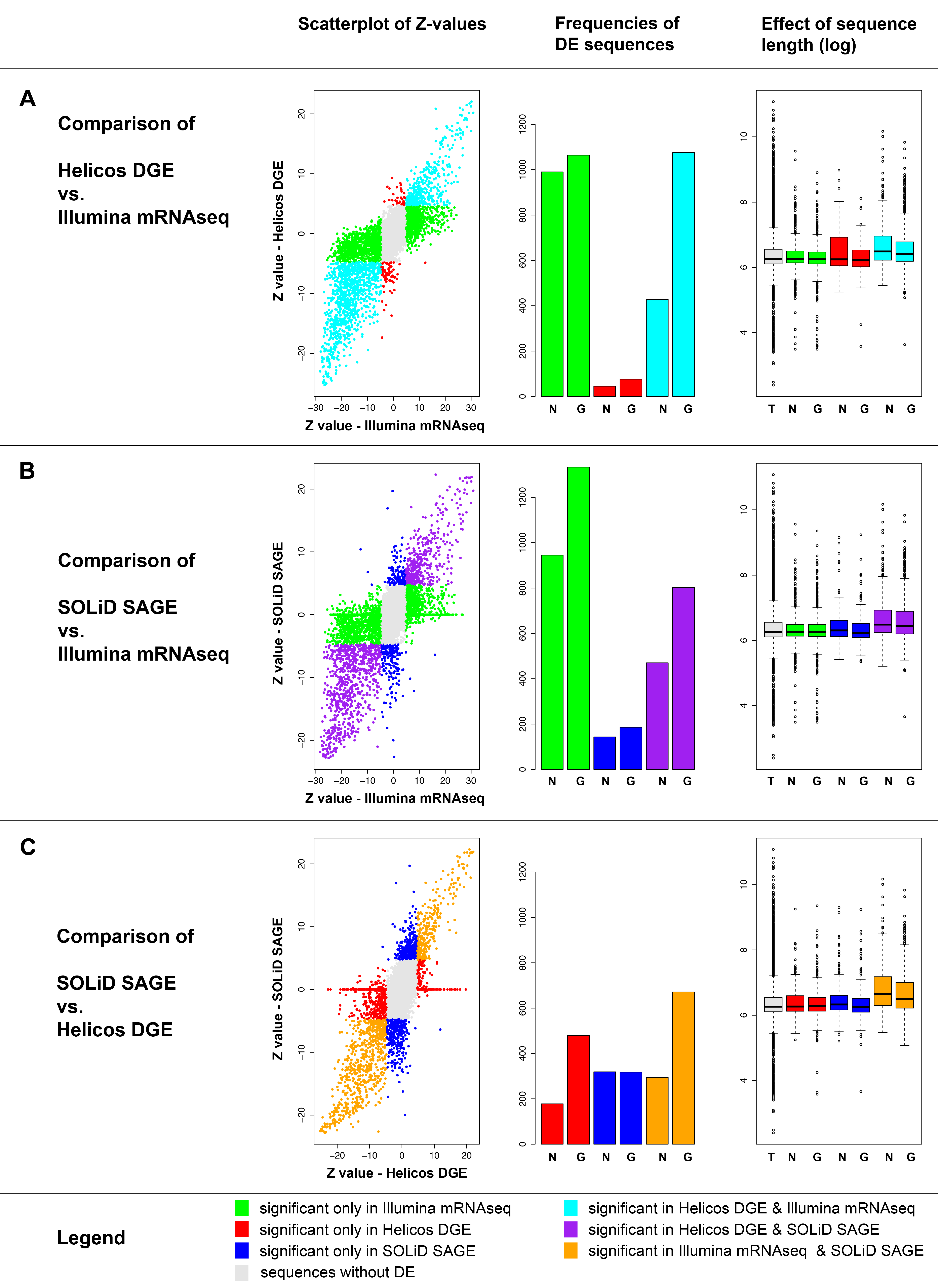

Supplement: Figure S7 — Congruence in detection of DE across sequencing workflows, considering all sequences in the reference. Visualized are scatterplots of pairwise comparisons (significance cuttoff Z>4.707364) (left), the frequency of DE in nectophores and gastrozooids for each category (middle) and DE in dependency of gene length. Positive Z values represent higher expression in nectophores, compared to gastrozooids (A) Helicos DGE – Illumina mRNA-Seq. (B) SOLiD SAGE – Illumina mRNA-Seq. (C) SOLiD SAGE – Helicos DGE. N: sequences indicated to be differentially expressed in nectophores, G: sequences indicated to be differentially expressed in gastrozooid, T: size distribution of all reference sequences. (PNG) [file pone.0022953.s007.png]

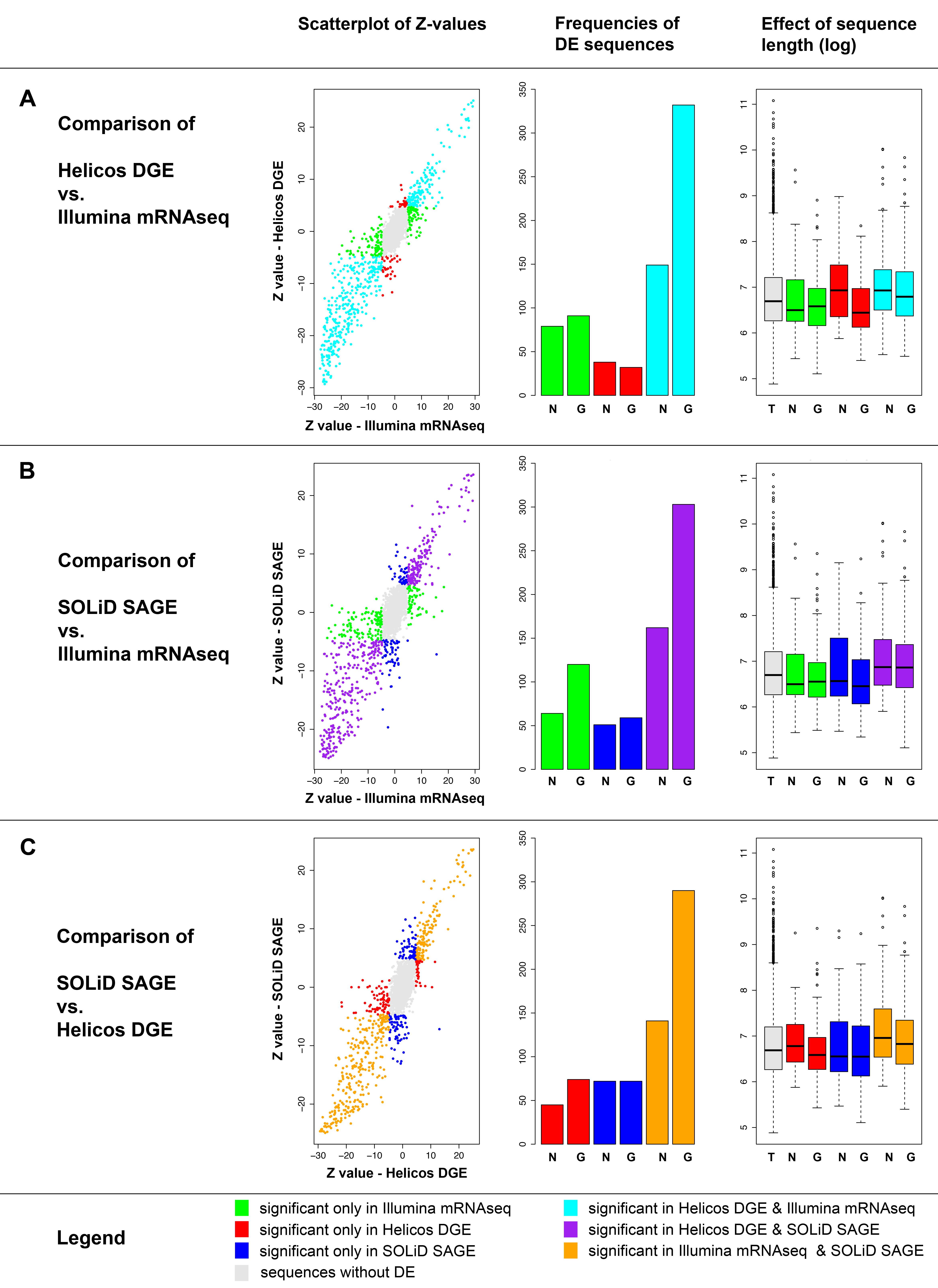

Supplement: Figure S8 — Congruences in detection of DE across sequencing workflows, considering the subset of reference sequences with the 3′-most NlaIII site. Visualized are scatterplots of pairwise comparisons (significance cutoff Z>4.382159) (left), the frequency of DE in nectophores and gastrozooids for each category (middle) and DE in dependency of gene length. Positive Z values represent higher expression in nectophores, compared to gastrozooids (A) Helicos DGE – Illumina mRNA-Seq. (B) SOLiD SAGE – Illumina mRNA-Seq. (C) SOLiD SAGE – Helicos DGE. N: sequences indicated to be differentially expressed in nectophores, G: sequences indicated to be differentially expressed in gastrozooid, T: size distribution of all reference sequences. (PNG) [file pone.0022953.s008.png]

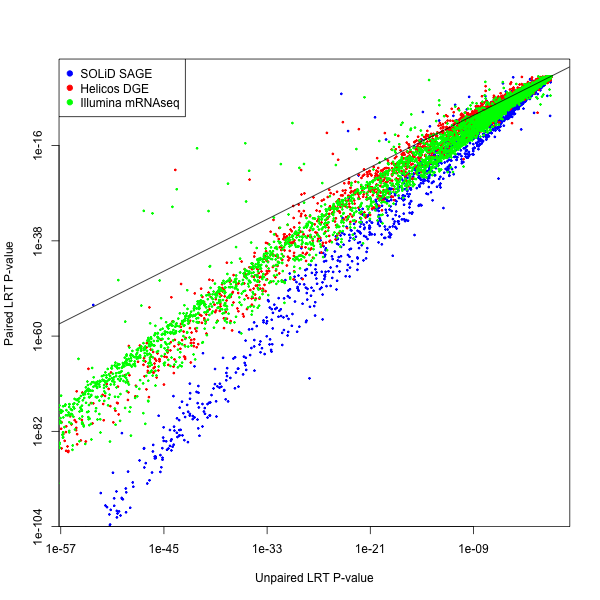

Supplement: Figure S9 — Variability of gene expression across field collected specimen of Nanomia bijuga . Comparison of likelihood ratio test p-values with or without considering that the samples are paired. Consistent p-values indicate low specimen-specific effects. (PNG) [file pone.0022953.s009.png]

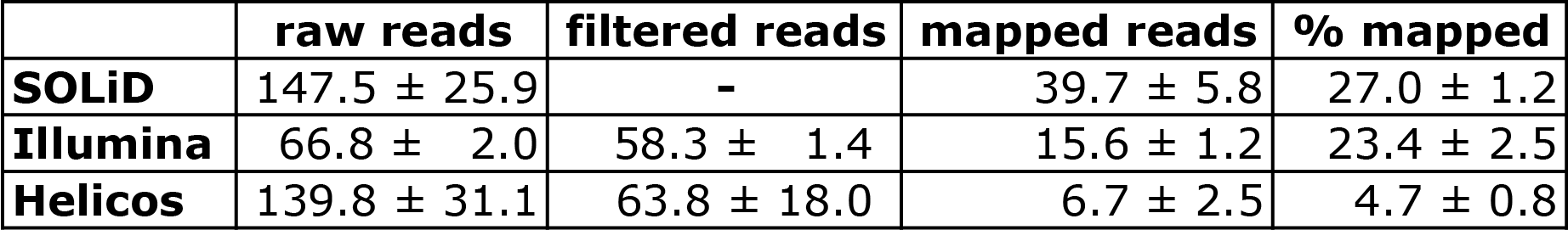

Supplement: Table S1 — Sequencing statistics for the three short-read platforms used to quantify gene expression in Nanomia bijuga . Shown are mean values and standard deviations for read numbers (in million reads) collected from three different animals. Percentage of mapped reads was calculated relative to raw reads. (PNG) [file pone.0022953.s012.png]
