# Supplementary material for: Differential Gene Expression in the Siphonophore Nanomia bijuga (Cnidaria) Assessed with Multiple Next-Generation Sequencing Workflows
Source: PLoS One. 2011 Jul 29;6(7):e22953. doi: 10.1371/journal.pone.0022953 (PMC3146525; doi:10.1371/journal.pone.0022953)

A **contig00076, gene=isogroup06489**

tblastx: DQ679808.1, "Hydra vulgaris fibrillar collagen (col3) mRNA, partial cds", e-value: 4.00E-88

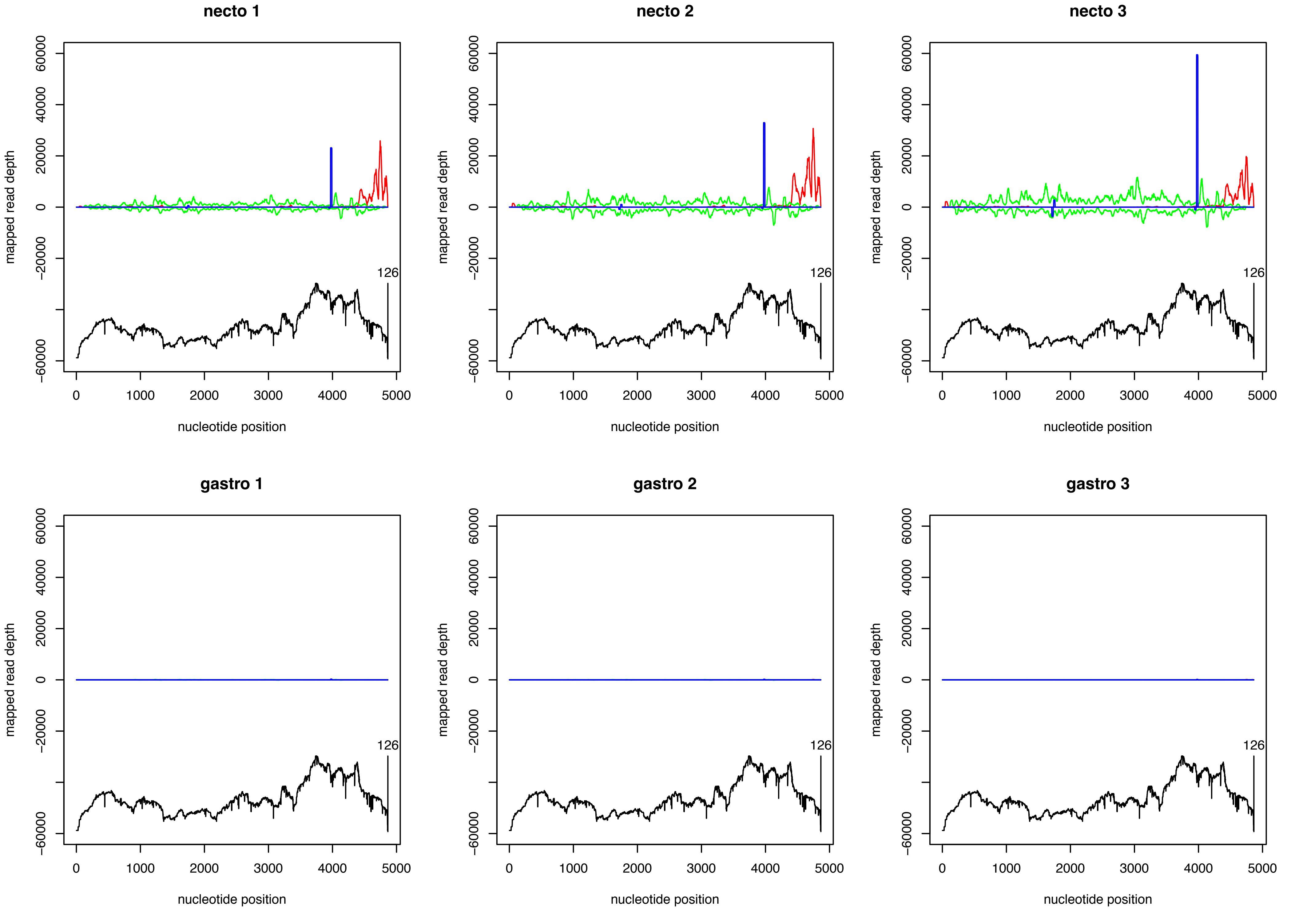

B **contig01067, gene=isogroup00259**
tblastx: XM_002169502.1, "PREDICTED: Hydra magnipapillata similar to GMP reductase 2 (LOC100202973)", e-value: 0.0

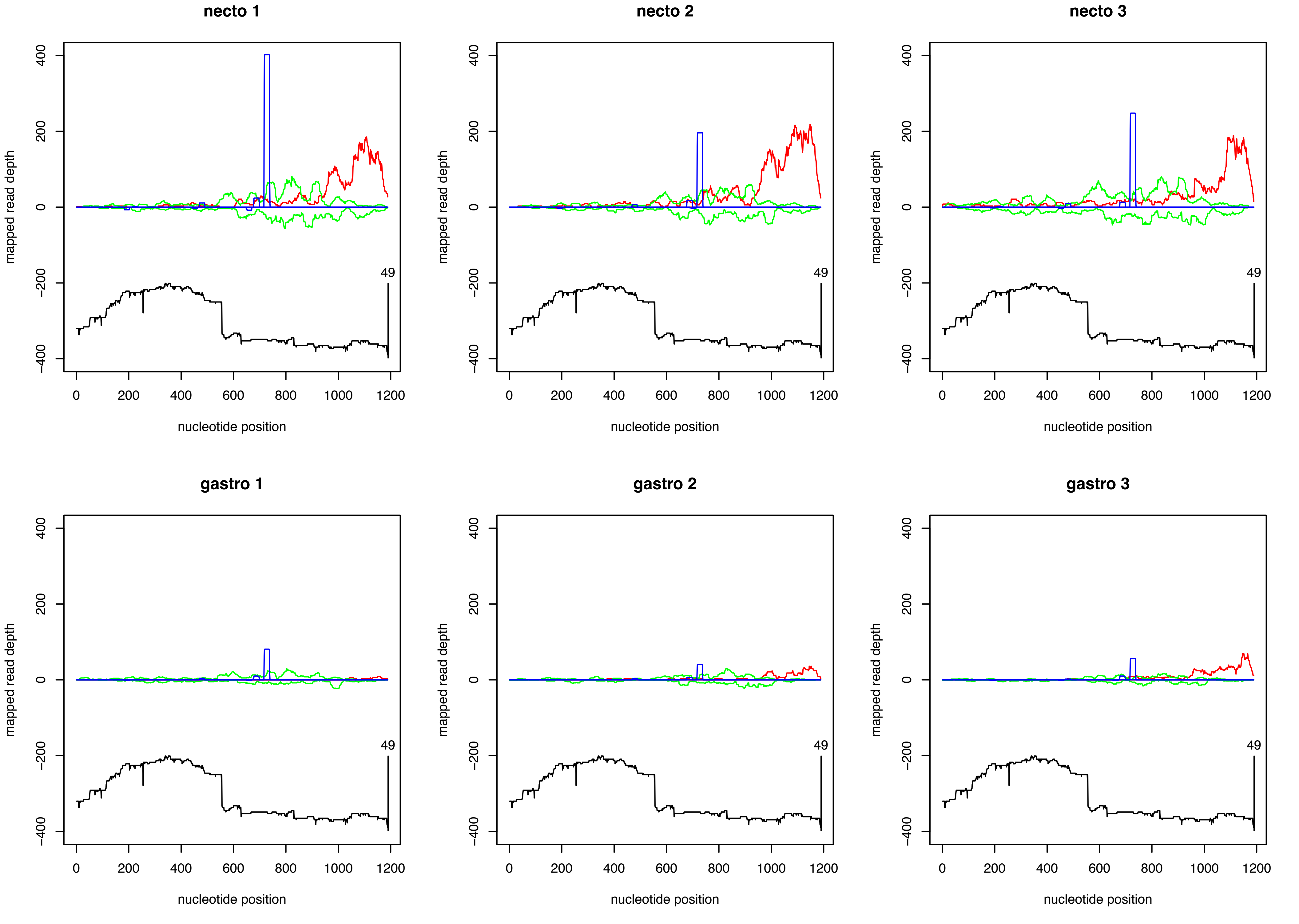

C **contig00101, gene=isogroup06398**
tblastx: XM_002196698.1, "PREDICTED: Taeniopygia guttata similar to alpha 2 type I collagen (LOC100221003), mRNA e-value: 2.00E-26

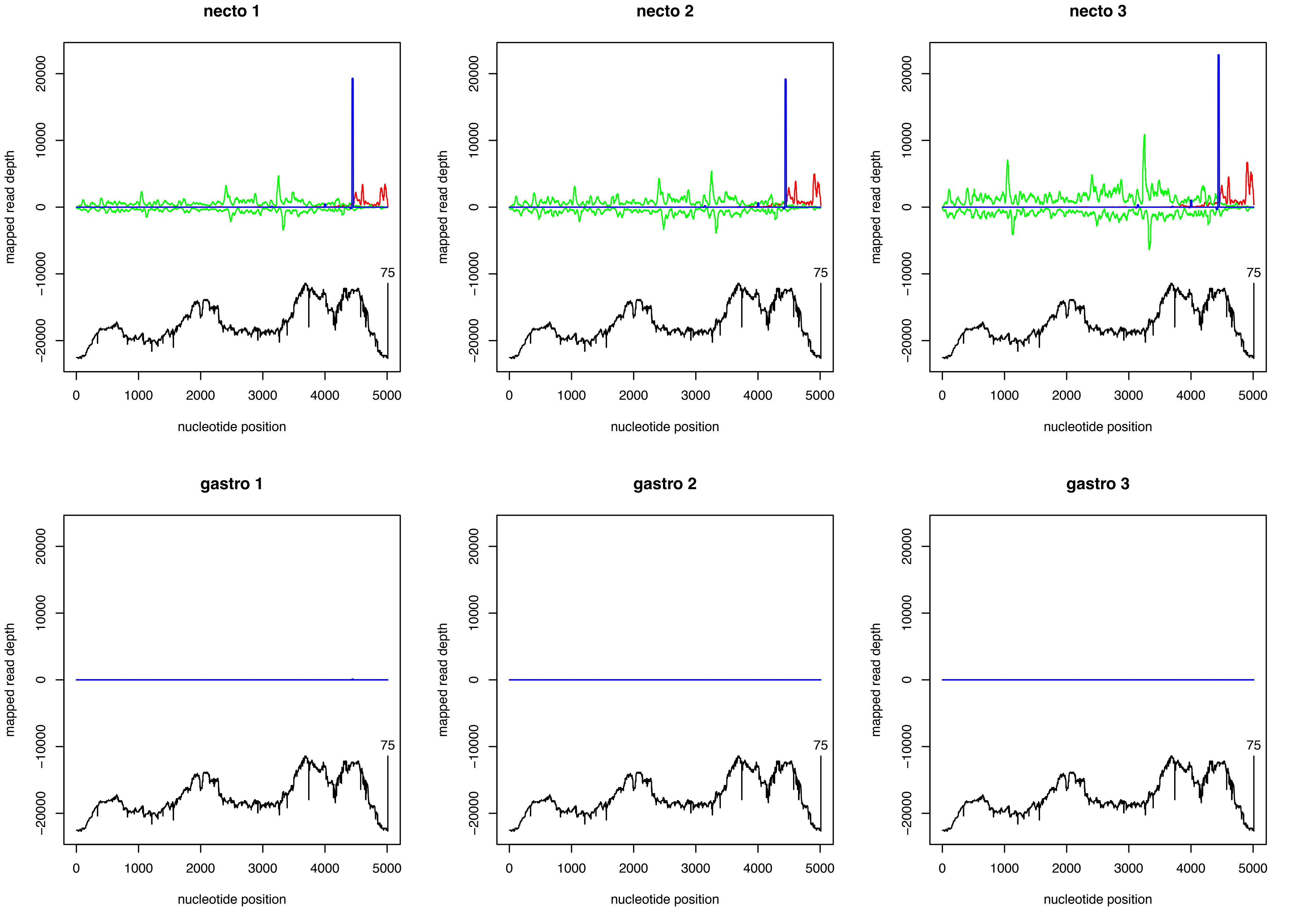

D **contig08699, gene=isogroup00303**

tblastx: XP_002157926.1, "PREDICTED: similar to myosin heavy chain [Hydra magnipapillata]", e-value: 0.0

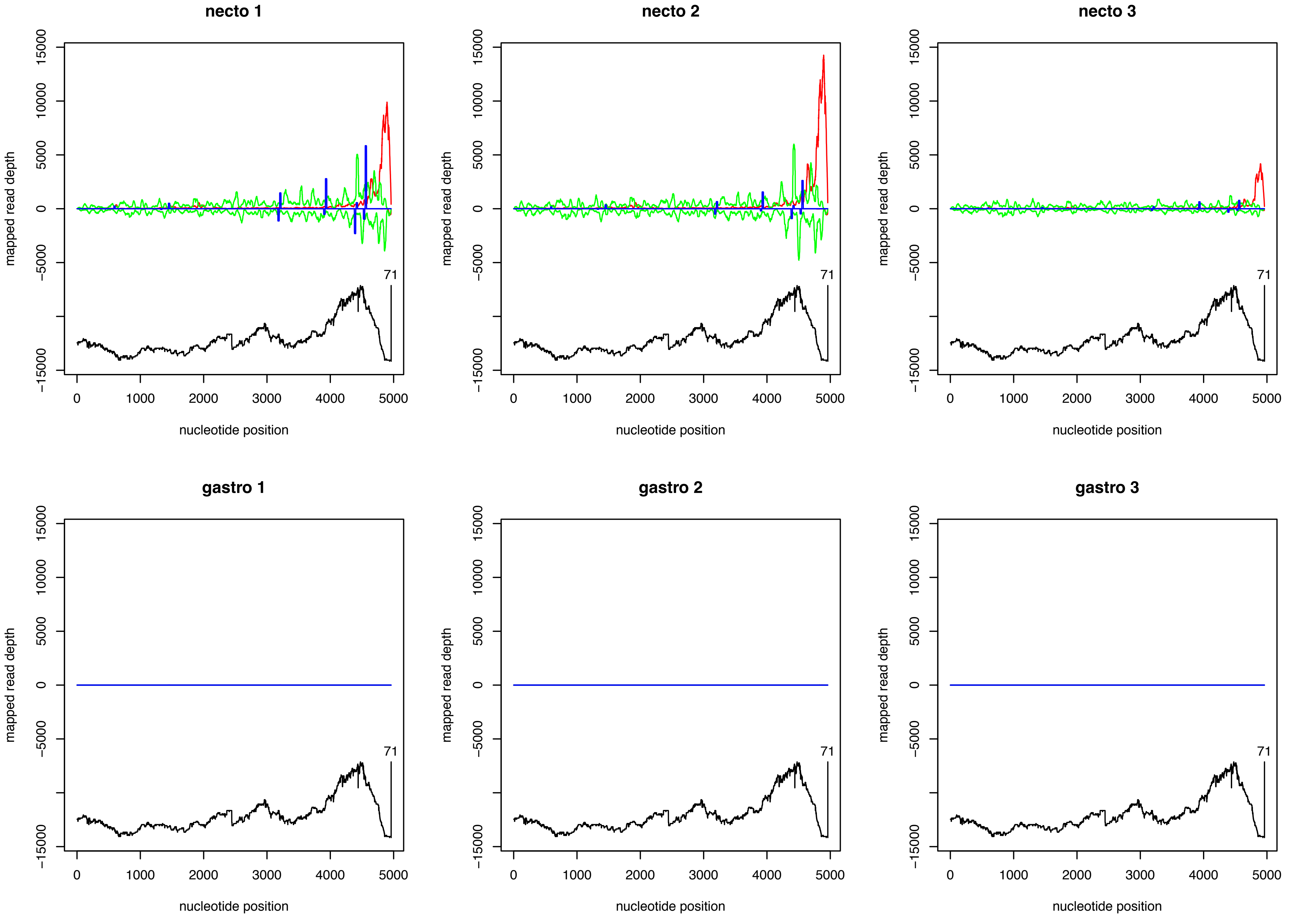

E **contig09291, gene=isogroup04804**

tblastx: XM_002166973.1, "PREDICTED: H. magnipapillata similar to predicted protein (LOC100209044), partial mRNA", e-value: 1.00E-19

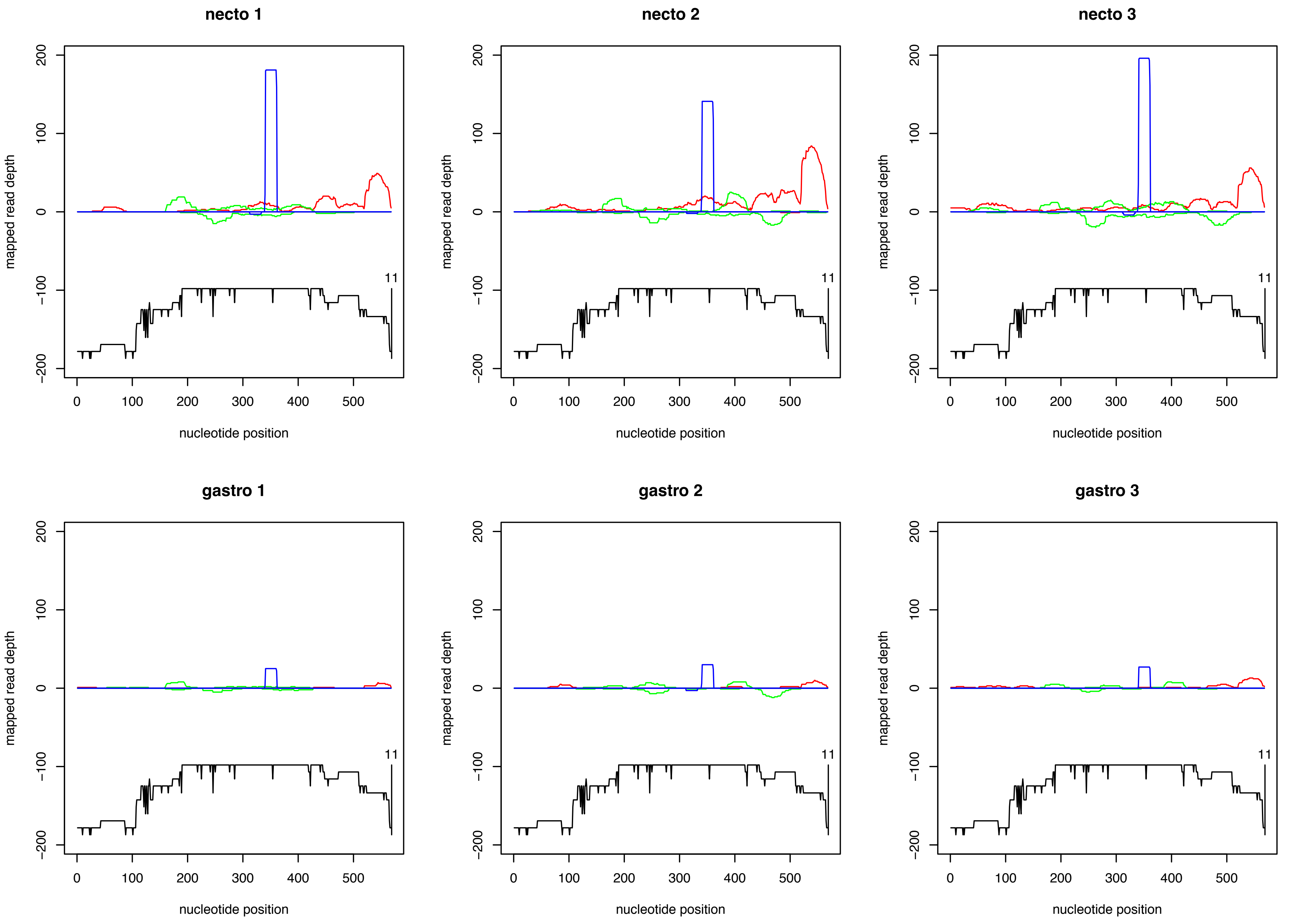

F **contig10617, gene=isogroup03256**
tblastx: XM_002158539.1, "PREDICTED: H. magnipapillata hypothetical protein LOC100210783, mRNA (Minicollagen)", e-value: 9.00E-12

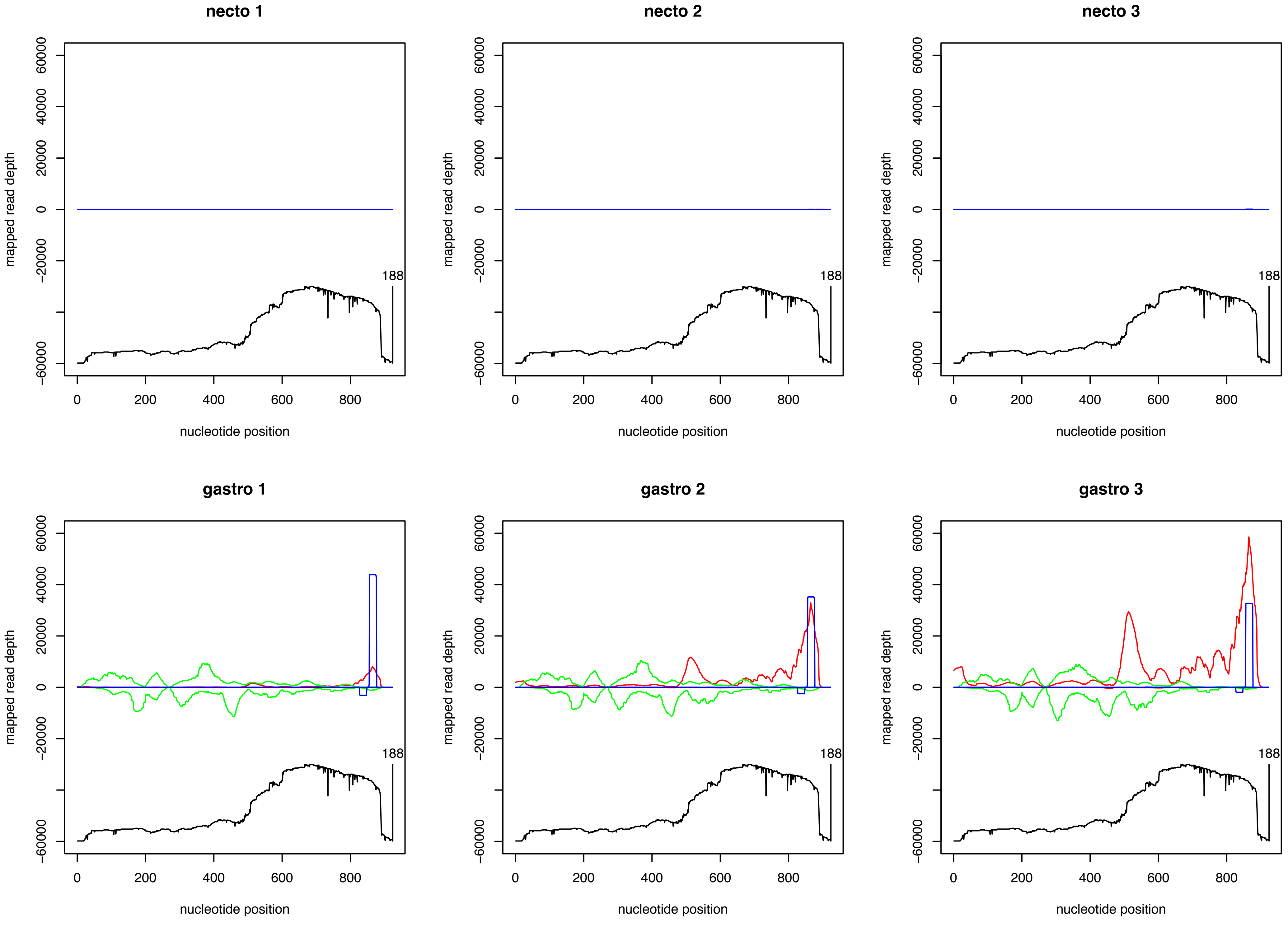

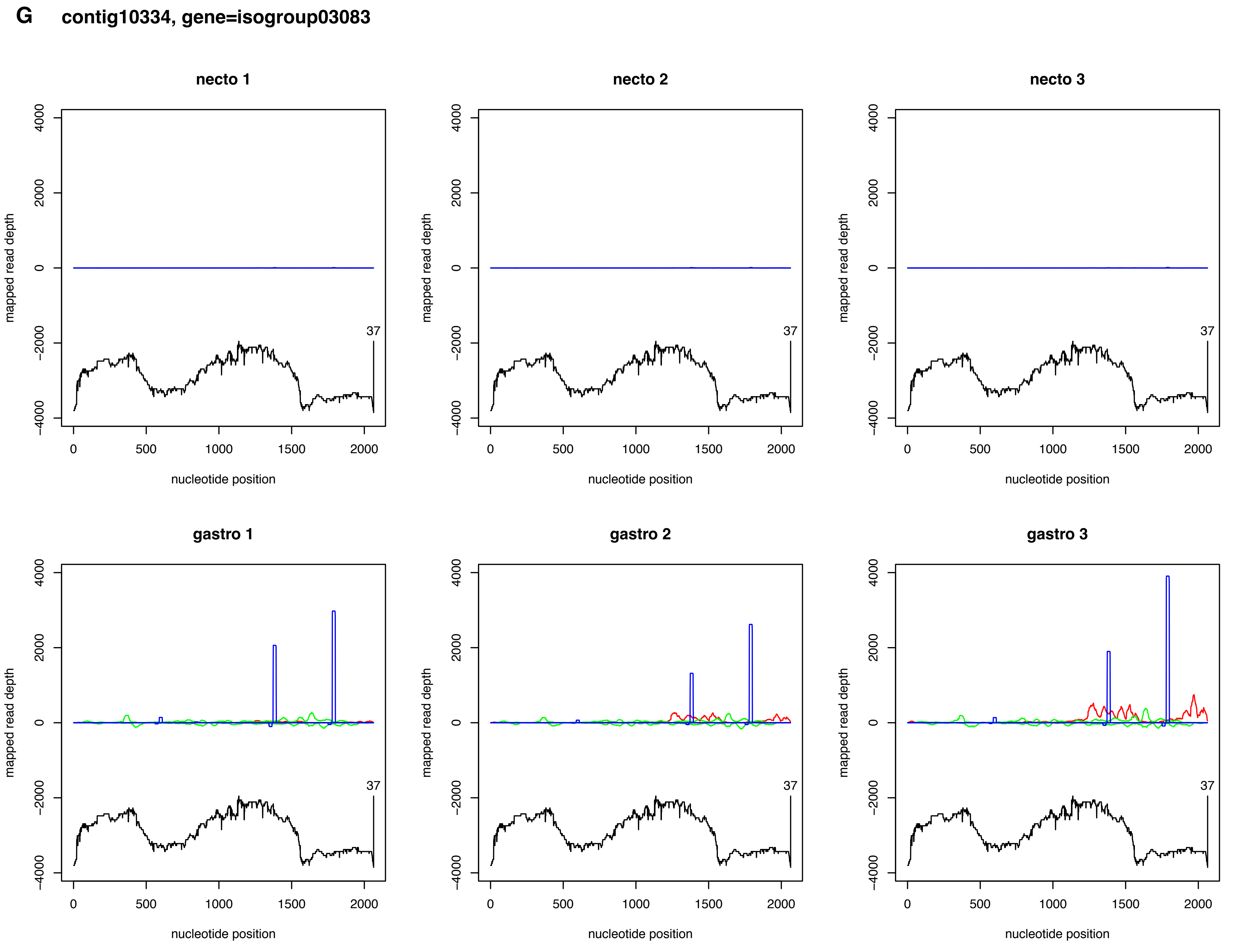

G
contig10334, gene=isogroup03083
necto 1
necto 2
necto 3
gastro 1
gastro 2
gastro 3
mapped read depth
nucleotide position
4000
2000
0
−2000
−4000
0
500
1000
1500
2000
37

H **contig04346, gene=isogroup07273**

tblastx: XM_002157903.1, "PREDICTED: H. magnipapillata similar to predicted protein (LOC100214126), partial mRNA", e-value: 6.00E-94

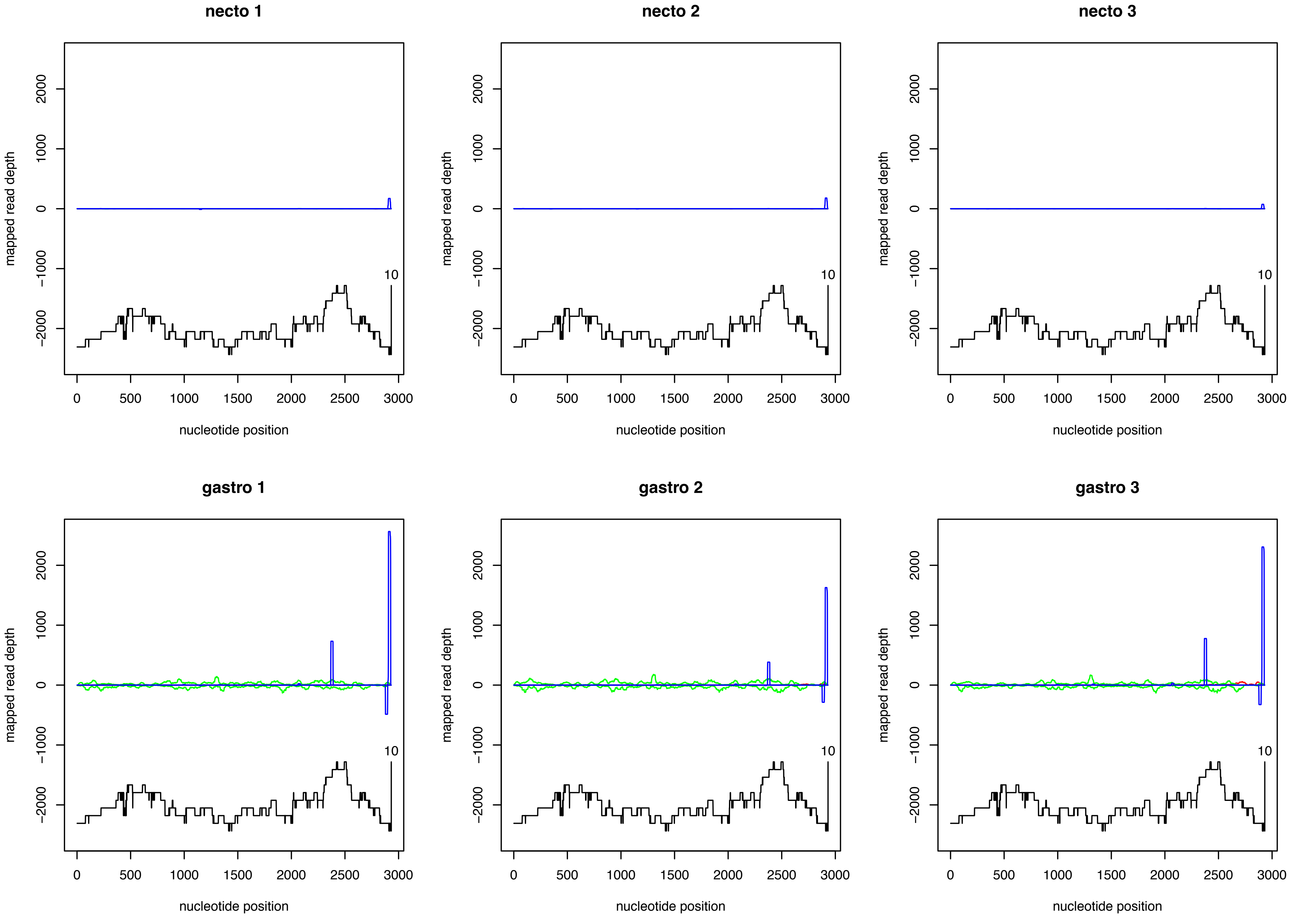

I contig04871, gene=isogroup07879

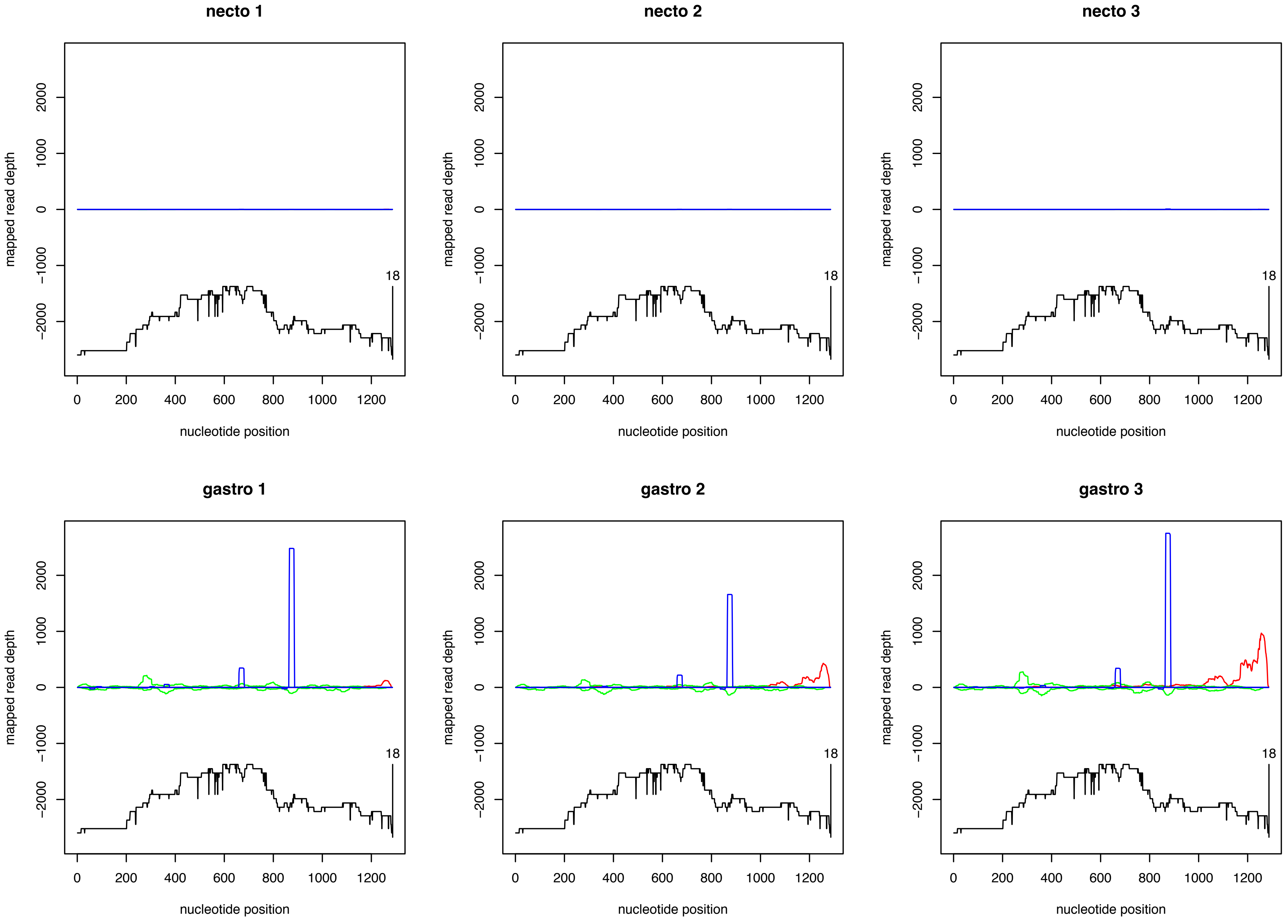

J **contig08098, gene=isogroup01314**

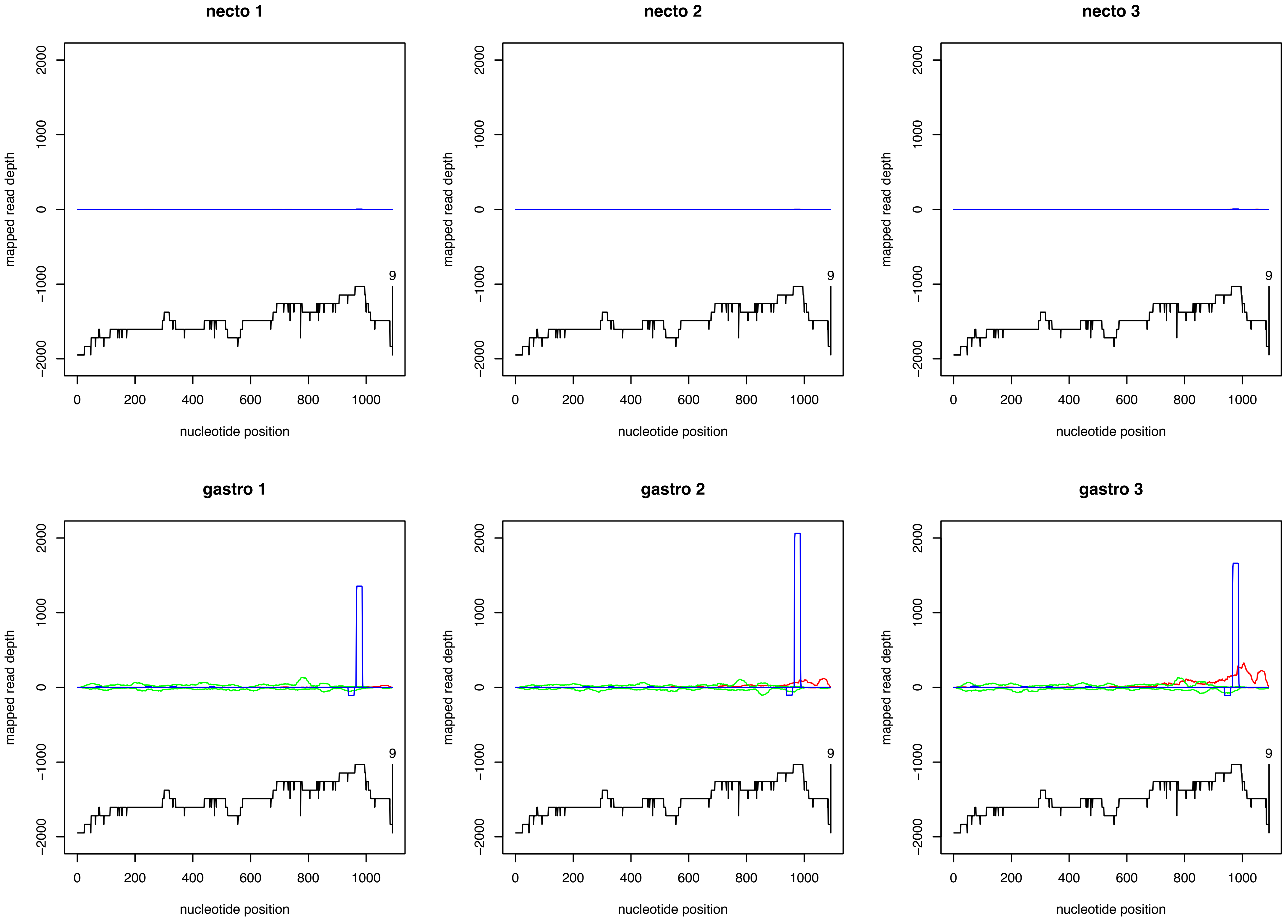

Supplement: Figure S6 — Mapped read distribution across selected transcripts. Read distribution is shown for ten different reference sequences (A–J) and all replicates (necto 1–3, gastro 1–3). Sequences are orientated in 5′ to 3′ direction. The physical distributions of mapped reads (non-normalized counts) across reference sequences were consistent for each platform across biological replicates. The given examples support the view of not exclusive but frequent ectopic read mapping of Helicos DGE reads to the 3′ end of the reference sequences. In each plot reads above the line map in the sense direction, below the line in the antisense direction. Helicos DGE reads (red) map to the sense strand, Illumina mRNA-Seq reads (green) map to sense and antisense strands along the whole reference sequence. The largest stack of reads for SOliD SAGE (blue) is adjacent to the 3′-most NlaIII cutting site. Height of the colored bars indicates the number of reads mapped to that location. 454 coverage at each nucleotide position of the reference sequence is shown in the lower part of each plot (scale bar indicates maximum depth of coverage in numbers of 454 reads). Best blast hit (tblastx against NCBI nr database) and respective e-value (e-value cutoff of 10−5) are given for each reference sequence if available. Plots in A–E show read distributions for nectophore specific transcripts and plots in F–J gastrozooid specific transcripts. Plots in A show read distributions (all replicates and both tissue types) for the fibrillar collagen (isogroup06489) also presented in Figure 2. Plots in F show read distributions for isogroup03256 (numbers of mapped SOLiD reads were downscaled by a factor of 100) which was further characterized by in situ hybridization (Figure 7). (PDF) [file pone.0022953.s006.pdf]
